# Topological data analysis quantifies biological nano-structure from single molecule localization microscopy

**DOI:** 10.1093/bioinformatics/btz788

**Published:** 2019-10-18

**Authors:** Jeremy A Pike, Abdullah O Khan, Chiara Pallini, Steven G Thomas, Markus Mund, Jonas Ries, Natalie S Poulter, Iain B Styles

**Affiliations:** 1 Centre of Membrane Proteins and Receptors (COMPARE), Universities of Birmingham and Nottingham, Midlands, UK; 2 Institute of Cardiovascular Sciences, College of Medical and Dental Sciences, University of Birmingham, Birmingham B15 2TT, UK; 3 Cell Biology and Biophysics Unit, European Molecular Biology Laboratory (EMBL), Heidelberg 69117, Germany; 4 Department of Biochemistry, University of Geneva, 1211 Geneva 4, Switzerland; 5 School of Computer Science, University of Birmingham, Birmingham B15 2TT, UK

## Abstract

**Motivation:**

Localization microscopy data is represented by a set of spatial coordinates, each corresponding to a single detection, that form a point cloud. This can be analyzed either by rendering an image from these coordinates, or by analyzing the point cloud directly. Analysis of this type has focused on clustering detections into distinct groups which produces measurements such as cluster area, but has limited capacity to quantify complex molecular organization and nano-structure.

**Results:**

We present a segmentation protocol which, through the application of persistence-based clustering, is capable of probing densely packed structures which vary in scale. An increase in segmentation performance over state-of-the-art methods is demonstrated. Moreover we employ persistent homology to move beyond clustering, and quantify the topological structure within data. This provides new information about the preserved shapes formed by molecular architecture. Our methods are flexible and we demonstrate this by applying them to receptor clustering in platelets, nuclear pore components, endocytic proteins and microtubule networks. Both 2D and 3D implementations are provided within RSMLM, an R package for pointillist-based analysis and batch processing of localization microscopy data.

**Availability and implementation:**

RSMLM has been released under the GNU General Public License v3.0 and is available at https://github.com/JeremyPike/RSMLM. Tutorials for this library implemented as Binder ready Jupyter notebooks are available at https://github.com/JeremyPike/RSMLM-tutorials.

**Supplementary information:**

Supplementary data are available at *Bioinformatics* online.

## 1 Introduction 

Single molecule localization microscopy (SMLM) is a super-resolution fluorescence imaging technique capable of localizing individual molecules to approximately 20 nm. Since its introduction (Betzig *et al.*, 2006; Hess *et al.*, 2006; Rust *et al.*, 2006), SMLM has matured as a technology and is now routinely used to probe biological nano-structure and processes for a range of biological applications (Leterrier *et al.*, 2015; Mund *et al.*, 2018). After performing localization, the data from a SMLM experiment is represented by a set of spatial coordinates, each corresponding to a single detection, that form a point cloud. This can be analyzed either by rendering an image from these coordinates and using image-based analysis methods, or by analyzing the point cloud directly. Strategies for the latter have focused on the concept of clustering, either by analyzing the spatial statistics of the point cloud to confirm the presence of clustered molecules (Owen *et al.*, 2010; Sengupta *et al.*, 2011; Veatch *et al.*, 2012), or by grouping individual detections into distinct clusters (Andronov *et al.*, 2016; Levet *et al.*, 2015; Owen *et al.*, 2010). This latter approach allows per-cluster statistics such as area and detection density to be calculated.

Clustering strategies commonly used for SMLM datasets estimate local detection density and construct clusters from the detections with density above a specified threshold. DBSCAN and Ripley’s K-based clustering estimate density using the number of neighbouring detections within a specified distance (Ester *et al.*, 1996; Owen *et al.*, 2010), whereas Voronoï diagram-based clustering uses the area of the tiles in the associated tessellation (Andronov *et al.*, 2016; Levet *et al.*, 2015). The free parameters, a density threshold and sometimes a distance scale, can be set manually or automatically using mean cluster density (Levet *et al.*, 2015) or Monte-Carlo simulations (Andronov *et al.*, 2016). If assumptions can be made about the distribution and shape of the clusters, a Bayesian engine can be used to set parameters (Rubin-Delanchy *et al.*, 2015). However, biological data is complex, often containing structures of significantly varying density. For such data a single density threshold is not sufficient and a multi-scale approach is required. Clustering algorithms can be repeated, using different parameter values, to segment structures at different densities, for example cells, organelles and protein clusters (Levet *et al.*, 2015). An alternative approach to density thresholding is to identify clusters based on persistence, or topographic prominence (Chazal *et al.*, 2013). This strategy has shown promise for SMLM datasets in the context of Ripley’s K-based clustering (Griffié *et al.*, 2015, 2017).

A further limitation of current clustering approaches is that topological information and higher order structure is not considered. Topological data analysis (TDA) provides a robust mathematical framework for probing the topology, or shape, of a point cloud. In this work we employ, and extend, methods from TDA, specifically persistence-based clustering (Chazal *et al.*, 2013) and persistent homology (Edelsbrunner *et al.*, 2002; Ghrist, 2007; Zomorodian and Carlsson, 2005), to quantify clustering and topological structure within SMLM datasets at a range of scales and densities. We demonstrate their ability to outperform existing methods and reveal new insight into biological nano-structure. Our clustering workflow is used to show a decrease in the area of platelet integrin α2β1 clusters when the tyrosine kinase Syk is inhibited. Additionally our persistent homology methodology is used to quantify topological structure for endocytic proteins and nuclear pore complex components. The tools are made available to the community as an R package.

## 2 System and results

### 2.1 Persistence-based clustering outperforms existing approaches

In common with many existing approaches, the first step in persistence-based clustering is the calculation of an underlying density estimate, *f* (Chazal *et al.*, 2013). Detections are assigned to local maxima within the density estimate by following the gradient of the density along a specified graph, a collection of points (detections) and lines linking pairs of points. This approach is known as mode seeking and facilitates the separation of clusters in close proximity (Koontz *et al.*, 1976). Mode seeking produces a collection of candidate clusters, or density modes. The maximum detection density within each candidate defines the birth density, *f_b_*, and the saddle point at which the candidate connects to a neighbouring candidate with higher birth density defines the death density, *f_d_* (Supplementary Fig. S1). For each candidate the persistence is defined as the difference between the birth density and the death density; $P=f_{b}-f_{d}$.

Finally a persistence threshold, *τ*, is specified and candidate clusters with P<τ are merged to a neighbouring candidate with P≥τ. Candidate clusters with P<τ which cannot be linked to a candidate with P≥τ are considered to be noise. This clustering scheme is named the Topological Mode Analysis Tool (ToMATo) and is analogous to local thresholding of the density estimate (Supplementary Figs S1 and S2). Further algorithmic details can be found in the Supplementary Methods and the original publication (Chazal *et al.*, 2013). A key advantage of ToMATo is the ability to segment clusters which are close together even if they vary in density.

**Fig. 1.**
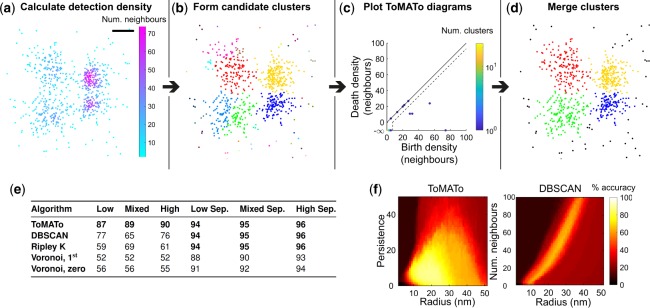
Persistence-based clustering for SMLM data. (**a**) 2D dSTORM simulation of four Gaussian clusters in close proximity with unequal variance (mixed density). The first step in the ToMATo algorithm is the calculation of detection density which is estimated by counting the number of neighboring detections within a fixed search radius, here set to the optimal value of 19 nm. Scale-bar 50 nm. (**b**) Detections are assigned to local maxima in the density estimate using a mode seeking approach. These density modes form candidate clusters. (**c**) ToMATo diagram showing the birth and death density for each candidate cluster. The birth density corresponds to the maximum detection density within the candidate, and the death density is the level at which the candidate merges to a stronger neighbouring cluster. The difference between the birth and death density defines the persistence of the candidate cluster and is represented on the diagram as the vertical distance from the diagonal. A persistence threshold is chosen below which clusters are merged (dotted line). Here this is set to the optimal value of 6 detections. The highest peak in each connected component resides at death density −∞. The colour bar represents the number of candidate clusters at a specified birth: death density, this is needed if more than more candidate is located at the same position on the diagram. (**d**) Final ToMATo clustering results after cluster merging. Noise detections, shown in black, are assigned when detections cannot be merged to a cluster above the persistence threshold. (**e**) Performance of clustering algorithms was quantified as the percentage of correctly assigned detections. Six different scenarios were simulated: low, mixed and high density clusters either in close proximity, or well separated (Sep.). For each scenario twenty simulations were analyzed and the maximal performance (averaged across simulations) for all parameter sets is shown. (**f**) Performance of ToMATo and DBSCAN across all tested parameters for the mixed density dataset

To evaluate ToMATo on SMLM data we generated realistic simulated dSTORM datasets of Gaussian clusters. Low, high and mixed (a mixture of high and low) density clusters were generated, either in close proximity or well separated (Supplementary Fig. S3). Local detection density was estimated by counting the number of detections within a fixed radius, and candidate clusters were constructed from the graph linking all detections within the same search radius (Fig. 1a and b). With our implementation of ToMATo there are two free parameters; the search radius and the persistence threshold, *τ*. To enable the selection of a suitable persistence threshold a scatter plot of the death and birth densities for each candidate cluster can be plotted, this is known as a ToMATo (persistence) diagram (Fig. 1c). Further guidance on parameter selection can be found in the Supplementary Methods.

We compared persistence-based clustering to existing routinely used approaches, specifically DBSCAN (Ester *et al.*, 1996), Ripley’s K-based clustering (Owen *et al.*, 2010; Rubin-Delanchy *et al.*, 2015) and Voronoï tessellation (Andronov *et al.*, 2016; Levet *et al.*, 2015). A range of free parameters were used for each simulation scenario and algorithm. Performance was quantified as the percentage of correctly assigned detections and averaged over repeated simulations (Supplementary Fig. S4). ToMATo significantly outperforms these existing approaches in challenging scenarios when clusters are close together (Fig. 1e). For the easier simulations where clusters are well separated it performs equally well. Moreover ToMATo is less sensitive to small changes in the choice of free parameters (Fig. 1f).

In biological applications it is common to have structures which are close together. These results demonstrate that persistence-based clustering is the highest performing and most stable of the tested algorithms for SMLM cluster analysis under these conditions. To better understand the sources of error for the ToMATo approach we classified detection assignment errors as either false positives, false negatives or incorrect cluster assignments (Supplementary Table S1). For scenarios where clusters were well separated the prevalent source of error was noise detections being assigned to clusters (false positives). This is because the overall error rate is low and randomly positioned noise detections which lie within the vicinity of clusters are not easily identified. As expected, when clusters are close together the prevalent source of error was incorrect cluster assignment.

To better understand the benefits and limitations of ToMATo clustering for SMLM data further simulations were performed where either the cluster separation, density, or signal to noise ratio (SNR) was varied (Supplementary Figs S5–S7). In the latter two cases clusters were well separated. For each algorithm a range of parameters were tested but fixed for the variations in cluster separation, density or SNR. When cluster separation was varied ToMATo outperformed the other tested algorithms with a large maximal performance increase of 36% at 60 nm cluster separation. When cluster density was varied ToMATo performed at least as well as the other tested algorithms. When the SNR was varied Ripley’s K-based clustering was the highest performing algorithm with a small maximal performance increase of 3.2% over ToMATo. This highlights a limitation of the ToMATo approach. If the SNR varies significantly within your data we recommend optimizing experimental conditions to reduce variation and pre-filtering detections based on density (Supplementary Fig. S7).

Persistence-based clustering is not limited to a particular structure, or shape, and is capable of segmenting non-circular objects such as microtubules, or even whole cells (Supplementary Fig. S8). However when working with fibrous structures specialized techniques such as those developed by Peters *et al.* (2017) should be considered.

### 2.2 Syk inhibition reduces the area of integrin α2β1 clusters in platelets

**Fig. 2.**
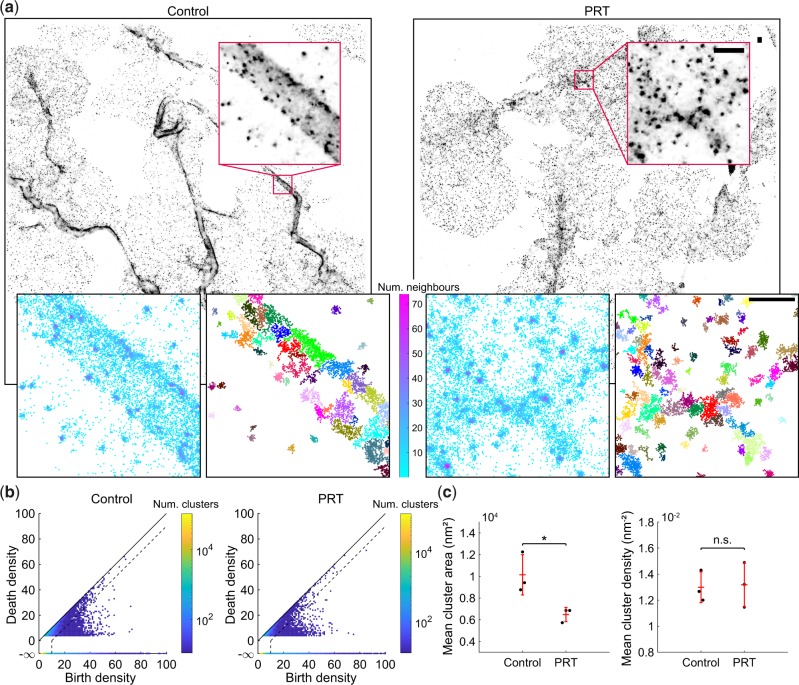
Syk inhibition reduces the mean area of integrin α2β1 clusters. (**a**) Platelets were seeded onto collagen fibres and treated with either the Syk inhibitor PRT060318, or a DMSO control. The sample was immunolabelled for integrin α2β1, secondary labelled with AlexaFluor647 and imaged using dSTORM. Persistence-based clustering (ToMATo) was used to segment integrin α2β1 nano-structures. Representative dSTORM image reconstructions, density estimates and clustering results (noise not shown). The search radius for the calculation of the density estimate and linking graph was set to 20 nm. Scale-bar 500 nm. (**b**) ToMATo diagrams showing the birth and death density for each candidate cluster. Dotted line shows the chosen persistence threshold for merging of clusters (10 detections). (**c**) Mean cluster area and cluster density. N = 3, four fields of view per replicate. The entire field of view was analyzed and mean cluster statistics were computed for all clusters in a replicate. Comparisons by two-sample *t*-test (*P <* *0.05), error bars are mean ± SD

To demonstrate the use of persistence-based clustering on real SMLM datasets we segment nano-structures of integrin α2β1 in platelets seeded on collagen fibres (Fig. 2a and b). Integrin α2β1, a platelet collagen receptor, accumulates at collagen fibres in spread platelets as shown in Supplementary Figure S9 (Poulter *et al.*, 2017). Within a single platelet there are areas with sparse or tightly packed α2β1 clusters due to differences in the underlying collagen distribution. This therefore represents a difficult multi-density segmentation problem. Stable platelet adhesion to collagen via α2β1 under flow conditions is dependent on the presence of the cytoskeletal adapter protein talin, which links integrins to the actin cytoskeleton (Beckerle *et al.*, 1989; Petrich *et al.*, 2007). We disrupted the cortical actin organization using the tyrosine kinase Syk inhibitor PRT060318 (Jaumouillé *et al.*, 2014; Reilly *et al.*, 2011), which we hypothesized would interfere with α2β1 clustering on collagen fibres. Syk inhibition results in a significant reduction in mean cluster area, however, no significant difference in cluster density was observed (Fig. 2c). A visual comparison with other segmentation methods is shown in Supplementary Figure S10 and computational time is compared in Supplementary Table S2.

### 2.3 Persistent homology quantifies topological nano-structure

SMLM data is information rich and contains more structural insight than is available through cluster analysis alone. Here we use persistent homology to extract complementary topological information from the data (Edelsbrunner *et al.*, 2002; Ghrist, 2007; Zomorodian and Carlsson, 2005). The concept of a graph can be extended to a higher dimensional structure, known as a simplicial complex. A simplicial complex is constructed from points, lines, triangles, tetrahedrons and equivalent higher order structures, collectively known as simplices. For SMLM data we build the simplicial complex on top of the point cloud formed by the detection list. There are a variety of methods for building complexes but this work focuses on the Rips complex, an abstract simplicial complex chosen for its efficient computation and storage. If each point within a candidate simplex (two points for a line and three for a triangle) is within a search distance of every other point, then the simplex is included in the complex. From the Rips complex the topological features of the underlying point cloud can be computed as a function of search distance, or scale. We will refer to this collection of features as the topological configuration. First order features correspond to the number of connected components in the complex, second order to the number of *holes* or *loops*. When working with 3D datasets, the third order features are enclosed *voids*.

**Fig. 3.**
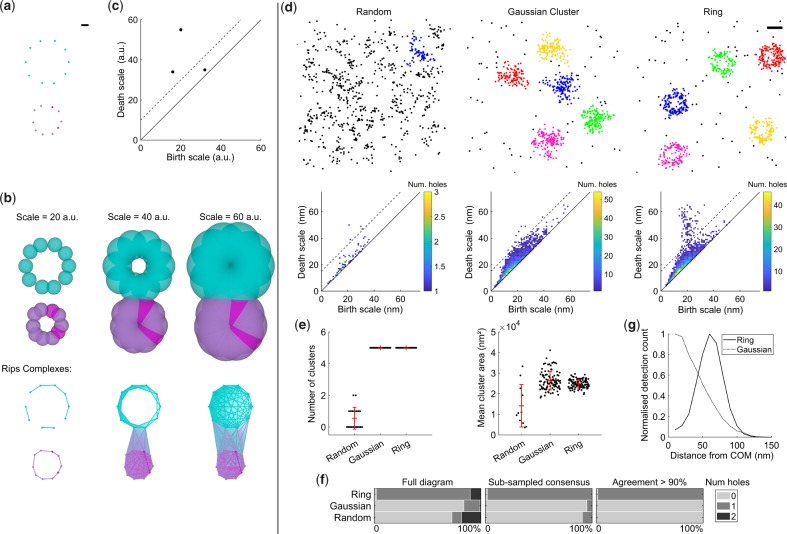
Persistent homology for topological analysis of clusters in SMLM datasets. (**a**) Illustrative example where detections are spaced evenly on the circumference of two circles. Scale-bar 10 arbitrary units (a.u.). (**b**) Building a filtration. Balls of varying diameter were placed at each detection (top) and the Rips complexes (bottom) were determined by the overlap of these balls. The filtration was evaluated for all integer values between 1 and 60 a.u. Without the filtration, it would be difficult to choose a scale which fully encapsulates the clustering and topology of the data. Simplex colour is set by the detection density estimate and is only for display purposes. (**c**) The persistence diagram summarizes structure within the filtration. The birth and death scales for each hole are shown. The persistence threshold is shown as a dotted line and there are two significant holes above this threshold. (**d**) Simulations for randomly distributed molecules, Gaussian clusters and rings with 60 nm radius were segmented using ToMATo. For each cluster a filtration was constructed and the corresponding persistence diagrams are shown. All holes have been grouped into a single persistence diagram per scenario. A persistence threshold of 15 nm was applied to all scenarios (dotted line). Scale-bar 100 nm. (**e**) Mean number of clusters and cluster area. Error bars are mean ± SD (**f**) Percentage of clusters with specified number of holes for each scenario. This was calculated using either the full diagram, the sub-sampled consensus, or the sub-sampled consensus with agreement > 90%. (**g**) Averaged radial distribution for clusters with agreement > 90%. As expected the peak of the profile for the ring simulation lies at 60 nm

Computing the topological structure within the data at a single scale is not very informative; any given feature could be unstable due to small variations in scale and it is not possible to capture multi-scale structure. To overcome this the Rips complex is computed for a range of scales, a process known as a filtration (Supplementary Video S1 and Fig. 3a and b). The birth scale of a feature is defined as the scale at which the feature forms in the filtration. Similarly, the death scale is defined as the scale at which the feature closes, or ceases. To summarize the information present in a filtration, the death scale and birth scale of each topological feature is plotted in a persistence diagram (Fig. 3c). The persistence of a feature is defined as the difference between the death and birth scales. The more robust a feature is to changes in scale, the greater it’s persistence, and the greater the distance from the diagonal of the diagram. Fragile features, typically noise, will have low persistence and be located close to the diagonal. Therefore thresholding features by persistence selects only the most robust, a procedure known as persistent homology (Edelsbrunner *et al.*, 2002; Ghrist, 2007; Zomorodian and Carlsson, 2005).

Before performing persistent homology it is advantageous to perform a cluster analysis to segment the data into nano-structures (Supplementary Fig. S11). This helps with interpretation of the results, and also reduces the computational cost (Supplementary Table S3). For each cluster a filtration is performed and a persistence diagram calculated. The number of features above the persistence threshold can then be counted for each cluster. SMLM data is inherently noisy and complex; containing labelling artifacts, significant localization uncertainty and substantial false positive rates. Persistent homology is robust to small perturbations in detection localization (Cohen-Steiner *et al.*, 2007) but not necessarily to the addition of noisy detections, or removal of true detections. To help overcome this we have developed an extension to the standard persistent homology workflow specific to SMLM. Instead of computing the persistence diagram for all detections within a cluster we sub-sample the cluster detections (with replacement) and repeatedly calculate the topological configuration for a specified persistence threshold. The sample size is set to the number of detections within the cluster. The sampling probability, *p*, can be weighted by detection measurements inherent to SMLM such as localization uncertainty; $p=e^{-\mu w}$. *w* is the normalized detection localization uncertainty, scaled between 0 and 1 for all detections within the cluster. *μ* is a constant which throughout this study is set to μ=−ln0.1=2.3. This was set such that the detection with the lowest localization uncertainty is ten times more likely to be sampled than the detection with the highest localization uncertainty. The most common configuration, the consensus and an agreement value, *α*, are returned. *α* corresponds to the percentage of sub-sampled clusters which return the consensus result and provides a confidence level for the specified topological configuration.

To test our persistent homology-based workflow for SMLM data, realistic synthetic datasets were generated. Molecules were distributed according to one of three scenarios; (i) complete spatial randomness (CSR), (ii) Gaussian clusters or (iii) circular rings with molecules evenly distributed on the circumference. We used ToMATo to segment the clusters and subsequently performed our persistent homology workflow to produce persistence diagrams (Fig. 3d and e and Supplementary Video S2). A single persistence threshold was applied to all scenarios and the number of holes for each cluster was calculated using both the full diagrams, and the sub-sampled consensus approach (Fig. 3f). Guidance for selecting appropriate persistence thresholds can be found in the Supplementary Methods and Supplementary Figure S12. The sub-sampled consensus approach produced significantly fewer incorrect classifications. Furthermore, by selecting only clusters with α>90% the percentage of incorrect classifications was reduced further. As expected no significant difference in cluster area was found between Gaussian and ring clusters. Therefore for these simulations standard cluster statistics cannot distinguish between Gaussian and ring clusters, whereas persistent homology clearly reveals the key topological difference. Several clusters were found in the CSR scenario due to artifacts such as multiple blinking events. However no CSR clusters with holes and α>90% were found demonstrating the robustness of our approach to noise.

The topology of clusters, as defined by our workflow, can be used to filter clusters for downstream analysis. For example we can select all clusters with a single hole (α>90%) and find the average radial distribution as shown for the ring simulations in Figure 3f. Our workflow can also be applied to 3D SMLM datasets as demonstrated for equivalent simulations in Supplementary Figure S13 and Supplementary Video S3.

### 2.4 Topological analysis of nuclear pore and endocytic proteins

To evaluate our novel methods on real data we focused on structures for which the topology has already been well characterized through image-based particle averaging. This is appropriate as it facilitates a robust validation of the proposed workflow. Specifically we choose Nup107, a component of the nuclear and cytoplasmic rings of nuclear pore complexes (Li *et al.*, 2018; Otsuka *et al.*, 2016; Szymborska *et al.*, 2013), and three different proteins of the yeast endocytic machinery; Las17, Ede1 and Sla1 (Mund *et al.*, 2018).

**Fig. 4.**
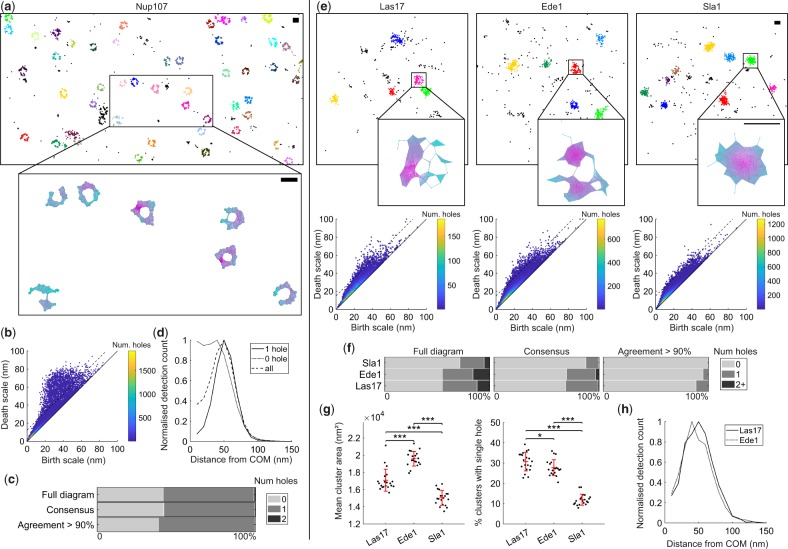
Persistent homology quantifies the topological configuration of biological nano-structures. (**a**) Topological analysis of Nup107-Snap-AlexaFluor647 imaged using dSTORM. Cropped field of view showing clustering result and example Rips complex evaluated at 50 nm. Scale-bar 100 nm. (**b**) A threshold of 15 nm was chosen for the persistence diagram (dotted line). (**c**) Percentage of Nup107 clusters with specified topological configuration. This was calculated using either the full diagram, the sub-sampled consensus, or the sub-sampled consensus with agreement > 90%. (**d**) Clusters were filtered for consensus agreement > 90% and the averaged radial distribution was plotted for clusters with either one hole, no hole or both. Peak for single hole clusters at 50 nm. (**e**) Topological analysis of the endocytic proteins Las17, Ede1 and Sla1 in yeast. Cropped fields of view showing clustering results and example Rips complexes evaluated at 30 nm. Persistence threshold was set to 15 nm (dotted lines). (**f**) Percentage of clusters with specified topological configuration. (**g**) Mean cluster area and the percentage of clusters with a single hole. Twenty fields of view were analyzed. To assess differences between endocytic proteins a one-way analysis of variance (ANOVA) was performed. If significant (*P *<* *0.05) subsequent pair-wise tests between endocytic proteins were performed using the Student’s *t*-test. *P* values were corrected for multiple comparisons using the Bonferroni method (*P <* *0.05, ***P <* *0.001). Error bars are mean ± SD (**h**) Averaged radial distributions for all clusters with a single hole and consensus agreement > 90%. Peak at 50 and 40 nm for Las17 and Ede1 respectively

Persistence-based clustering was used as a pre-processing step to segment either the nuclear pore complex, or endocytic sites (Supplementary Figs S14–S16). For Nup107 both 2D and 3D dSTORM datasets were analyzed. For the 2D Nup107 dataset the resulting persistence diagram shows a large number of holes above the threshold (Fig. 4a and b). As expected a large proportion of the clusters have a single hole topology (Fig. 4c). However even for the sub-sampled approach, with α>90% filtering, a significant proportion of clusters (39%) are found to have no holes. These clusters could be a result of many scenarios including clustered molecules not in a pore complex, imaging artifacts, unspecific labelling and variation in pore alignment. Filtering of clusters with a single hole results in an average radial profile with a peak at 50 nm, reproducing the results of imaged-based particle averaging (Fig. 4d) (Szymborska *et al.*, 2013). No radial structure is apparent when clusters with no holes are averaged. This implies these clusters are correctly assigned by the persistent homology workflow.

When imaged in 3D both the cytoplasmic and nuclear rings of the complex must be considered. Both rings should have the topology of a cylinder and contain a single hole and no enclosed voids. When our method is applied to 3D data we observe large numbers of structures with no topological features (81%). However a significant number of clusters have single holes (17%) and a small percentage have two holes (1%) (Supplementary Fig. S16). No structures had enclosed voids. The quoted percentages are for the sub-sampled approach with α>90% filtering.

In a recent study the actin nucleation promoting factor Las17, was shown to have a clear ring profile when many endocytic sites are averaged, whereas the coat protein Sla1 does not (Mund *et al.*, 2018). It was also shown that Ede1 is recruited to sites in the early stages of endocytosis, and clusters are not uniform in size and shape, hence a ring like topology is not clear from image-based particle averaging without filtering of clusters. We take data from this study, where endocytic proteins in yeast were endogenously labelled with a photoconvertible fluorescent protein and imaged using a homebuilt SMLM system, and apply our novel topological analysis workflow. Figure 4e shows example cluster results and persistence diagrams for all three proteins. From visual inspection of the diagrams it is clear that both Las17 and Ede1 clusters have large numbers of holes, whereas Sla1 clusters do not. Quantification of the percentage of clusters with holes reveals a significant difference between all three proteins with Las17 having the highest percentage of single hole clusters and Sla1 the lowest (Fig. 4f and g). The averaged radial profile of single hole clusters (α>90%) for Las17 and Ede1 have a clear ring structure with maximum at 50 and 40 nm respectively, confirming the results of imaged-based analysis (Fig. 4h). Together these results demonstrate that our method is able to accurately and efficiently quantify nano-scale topology in SMLM datasets.

## 3 Implementation

### 3.1 RSMLM: R package for analysis of SMLM datasets

To complement this study we have released RSMLM, an R package for the pointillist-based analysis of SMLM data. This package includes the methods described in this paper for persistence-based clustering (ToMATo), alongside DBSCAN, Voronoï tessellation and Ripley’s K-based clustering. Utility methods for persistent homology including the sub-sampling consensus approach are provided. There is also the capacity to simulate dSTORM data. This library will provide an adaptable framework for analysing and batch processing both 2D, and 3D, SMLM datasets. Binder ready Jupyter notebook tutorials are provided to facilitate easy use of the package. The functionality of the library can also be included within KNIME workflows using simple R-snippets (Berthold *et al.*, 2009). This enables users without any scripting knowledge to access the core functionality.

### 3.2 Persistence-based clustering

Persistence-based clustering, or ToMATo, was originally developed by (Chazal *et al.*, 2013) and implemented for this work as follows. Detection density estimates were calculated by counting the number of other detections within a specified radius. This was implemented using the R package dbscan which uses the C++ library ANN to employ a k-d tree framework for efficient computation. Density modes were calculated using a mode seeking approach and a Rips graph Koontz *et al.* (1976). Modes were then merged or designated as noise based on a specified persistence threshold. This was implemented by adapting C++ scripts (GPLv3) described in Chazal *et al.* (2013). C++ functions were incorporated into RSMLM using the R package Rcpp. For algorithm comparison the search radius ranged from 1 to 50 nm, and the persistence threshold from 0 to 50 detections. Further algorithmic details and the implementations of other clustering algorithms are described in the Supplementary Methods.

### 3.3 Persistent homology

Rips filtrations and persistence diagrams were computed using the R package TDA which employs the C++ library GUDHI (Fasy *et al.*, 2014; Maria *et al.*, 2014). When the density of points in a persistence diagram was too high for a simple plot, features were grouped into a joint histogram with 1 nm^2^ bins and displayed using a heat-map. Visualizations of Rips complexes were created using plex-viewer (https://github.com/atausz/plex-viewer) and the Java library javaPlex (Adams *et al.*, 2014). Filtration movies were created using POV-Ray.

### 3.4 dSTORM simulations

Fluorophore blinking characteristics were modelled using a geometric distribution with probability of transition to the dark state set to 0.5 (Lee *et al.*, 2012). Simulated molecules were bound to an average of 5 fluorophores, randomly distributed between molecules. The localization uncertainty for each blinking event was determined using a normal distribution centred on the molecule position. Standard deviation for localization uncertainty was set using a log-normal distribution with mean 2.8 and standard deviation 0.28 (experimentally measured parameters for AlexaFluor 647) (Mollazade *et al.*, 2017). Detection rate for blinking events was set to 70%. 10% false detections (noise) were added and distributed randomly across the field of view. Further simulation parameters are described in the Supplementary Methods.

### 3.5 Real datasets

Methodological details for the dSTORM imaging of integrin α2β1 in platelets is described in the Supplementary Methods. The 2D and 3D SMLM Nup107 data was recently published and described in (Li *et al.*, 2018) where Nup107–SNAP Alexa Fluor 647 was imaged using dSTORM in U-2 OS cells. 3D localization was achieved using an experimental PSF model. SMLM datasets for Las17, Ede1 and Sla1 were recently described in (Mund *et al.*, 2018). In short endocytic proteins were endogenously tagged with the photoconvertible protein mMaple (McEvoy *et al.*, 2012) in budding yeast strains and imaged in high-throughput using a homebuilt SMLM imaging system (Deschamps *et al.*, 2016). Image acquisition for *α*-tubulin dSTORM was performed for formalin fixed A549 cells immunofluorescence labelled for *α*-tubulin (Alexa-647 secondary) as previously described in Khan *et al.* (2017). Detections in the integrin α2β1, *α*-tubulin and Nup107 datasets were filtered by intensity with a minimum value of 1000 photons. For all datasets detections that were found in consecutive frames within a distance of 75 nm were grouped into a single detection.

## 4 Discussion

As single molecule methods for high throughput data acquisition (Beghin *et al.*, 2017) and sample labelling (Khan *et al.*, 2017) improve, there is a parallel need for automated, robust analytical approaches. The mathematical field of topological data analysis provides a powerful framework for structural analysis of SMLM data. We have introduced tools using both persistence-based clustering (Chazal *et al.*, 2013) and persistent homology (Ghrist, 2007) to quantify biological nano-structure. Empirical evidence suggests ToMATo provides superior clustering performance over commonly used algorithms for SMLM data when clusters are close together, even if clusters vary in density (Andronov *et al.*, 2016; Ester *et al.*, 1996; Levet *et al.*, 2015; Owen *et al.*, 2010). We argue that biological nano-structure is rarely simple and for many applications these conditions will be commonplace, as we show for clustering of collagen receptors in platelets. Moreover selection of free parameters is less sensitive for persistence-based clustering, and can be guided by ToMATo diagrams. Whilst Bayesian approaches (Rubin-Delanchy *et al.*, 2015) to parameter setting are very powerful when the underlying nano-structure can be modeled in advance, it is not suitable for exploratory applications where the nano-structure is not known *a priori*.

A disadvantage of the ToMATo approach when compared to Voronoï tessellation-based clustering is that there are two free parameters to set, whereas tessellation only has one which can be normalized. The core concepts of this work could also be transferred to other clustering approaches which could be extended to threshold on persistence (Griffié *et al.*, 2015, 2017). For example Voronoï tessellation could be used to calculate the density estimate. The dual graph of the tessellation could also be used to link detections resulting in an algorithm with only one free parameter, providing an interesting avenue for further research.

Persistent homology is designed to reveal topological structure within pointillist datasets and has natural applications for the analysis of SMLM data, as also demonstrated in parallel work (Hofmann *et al.*, 2018). Our framework moves beyond per cluster statistics such as area and density to reveal new information about the underlying topological features within segmented nano-structures. Importantly, this is a multi-scale approach, revealing unbiased structural information at a range of distance scales simultaneously. Furthermore we have developed a novel extension of the standard persistent homology workflow which is based on sub-sampling of detections. This extension ensures the method is robust to SMLM artifacts and provides a confidence value for any specific result. Validation of these approaches was performed using nuclear pore and endocytic site proteins. Similar structural information can be gained from SMLM data by image-based particle averaging (Mund *et al.*, 2018; Szymborska *et al.*, 2013). However persistent homology can be used in cases where particle averaging methods may fail, for example, where the underlying structures vary in shape or size but maintains a constant topology. Persistent homology can also quantify the structure of individual clusters, not just average ensembles, and is suitable for studying smaller datasets. Finally image-based analysis workflows are typically complex with many pre-processing steps specific to the application. Our streamlined workflow requires minimal adaptation for different datasets as demonstrated throughout this study.

Our methods for persistence-based clustering and persistent homology have been implemented and validated in both 2D and 3D. This framework is applicable for a wide range of problems, revealing new topological information and unprecedented insight into biological nano-structure not attainable from existing tools.

## Supplementary Material

btz788_Supplementary_Data
