# Supplementary material for: Topological data analysis quantifies biological nano-structure from single molecule localization microscopy
Source: Bioinformatics. 2019 Oct 18;36(5):1614–21. doi: 10.1093/bioinformatics/btz788 (PMC7162425; doi:10.1093/bioinformatics/btz788)
Supplement: btz788_Supplementary_Data [file btz788_supplementary_data.zip › btz788-Suppl_Data/supplementary.pdf]

Supplementary Note 1: Materials and Methods

Topological Mode Analysis Tool (ToMATo) algorithmic details. The ToMATo clustering algorithm was originally proposed by (Chazal *et al.*, 2013) and the interested reader should refer to this original work for a detailed technical description. Here we briefly summarise. Given a set of detections, $\mathbf{p} = \mathbf{p}_1, ..., \mathbf{p}_n$, we define a function, $f : \mathbf{p} \rightarrow \mathbb{R}$, which estimates the local density for each detection. For this work we simply count the number of detections within a set distance, r, such that;

$$f(\mathbf{p}_i) = \sum_{j=1}^{n} I(d_{ij} \leq r) \tag{1}$$

where I is the indicator function and d_{ij} is the euclidean distance between detections $\mathbf{p}_i$ and $\mathbf{p}_j$ (**Supplementary Fig. 1a,b** and **Supplementary Fig. 2a**). The positive constant, r, is the first free parameter of the algorithm. It what follows we assume $\mathbf{p}$ has been sorted such that $f(\mathbf{p}_1) \geq f(\mathbf{p}_2) ... \geq f(\mathbf{p}_{n-1}) \geq f(\mathbf{p}_n)$. Next the Rips graph, $G(\mathbf{p})$, is formed by linking all detections where $d_{ij} \leq r$ (**Supplementary Fig. 2b**). The remaining algorithm is broken into two steps:

1. **Form candidate clusters by mode seeking.** Let $\mathbf{q}_{ij}$ be the neighbours of $\mathbf{p}_i$ within G and let $\mathbf{q}_i^{\max} \in \mathrm{q}_{ij}$ be the neighbour with the highest density (**Supplementary Fig. 2b**). Iterate through the sorted detections and link $\mathbf{p}_i$ to $\mathbf{q}_i^{\max}$ when $f(\mathbf{q}_i^{\max}) \geq f(\mathbf{p}_i)$. If $f(\mathbf{q}_i^{\max}) < f(\mathbf{p}_i)$ then $\mathbf{p}_i$ is a peak and defines the birth density of a new candidate cluster. The result is a spanning forest of G where each tree corresponds to a candidate cluster (**Supplementary Fig. 1c**).

2. **Merge clusters based on persistence.** Iterate through the sorted detections, $\mathbf{p}_i$, and the neighbouring detections, $\mathbf{q}_{ij}$. Let α and β be the clusters containing $\mathbf{p}_i$ and $\mathbf{q}_{ij}$ respectively and let $f_b(\alpha)$ and $f_b(\beta)$ be the birth densities of these clusters. If $f(\mathbf{p}_i) \leq f(\mathbf{q}_{ij})$ and $f_b(\beta) < \min\{f_b(\alpha), f(\mathbf{p}_i) + \tau\}$ then clusters α and β are merged (**Supplementary Fig. 2c,d**). τ is the persistence threshold and the second free parameter of the algorithm.

To calculate the ToMATo diagram the merging step is run with $\tau = \infty$ and death densities, f_d, are defined as the density, $f(\mathbf{p}_i)$, at which clusters merge.

Other clustering algorithms. Density based spatial clustering of applications with noise (DBSCAN) was implemented using the R package dbscan (Ester *et al.*, 1996). Edge detections were included in clusters. For algorithm comparison the search radius ranged from 1 to 50 nm, and the density threshold from 1 to 100 nm.
Ripley K based clustering was implemented by finding the number of detections within a specified search radius, r, and using this to calculate the Ripley L function, $L(r)$, for each detection. $L(r) - r$ was subsequently used as a density estimate and thresholded (Owen *et al.*, 2010). Filtered detections were grouped into clusters by finding the connected components of the graph formed by linking all filtered detections within r (Rubin-Delanchy *et al.*, 2015). For algorithm comparison the search radius, r, ranged from 1 to 50 nm, and the $L(r) - r$ threshold from -10 to 30.
Voronoï tesselations were calculated using the R package deldir. Tesselation based clustering was implemented by removing all tiles where the normalised detection density was below a specified threshold. Density was either defined as the inverse of tile area (Voronoï zero) (Andronov *et al.*, 2016), or the inverse of mean first rank tile area (Voronoï 1st) (Levet *et al.*, 2015). Mean first rank tile area is defined as the mean area of the specified tile and all adjacent tiles. Density estimates were normalised by dividing by the mean density across the full field of view. After removing tiles from the tessellation, clusters were formed from the connected components of the dual graph (the Delaunay triangulation). For algorithm comparison the normalised density thresholds ranged from 1 to 5.

Sub-sampled persistent homology. After segmenting individual clusters, or structures, detections were sampled from each cluster with replacement (bootstrapping). Throughout this study the number of iterations was set to 50 and the sample size was set to the number of detections in the cluster. The sampling probability, p, was weighted such that $p = e^{-\mu w}$, where w is the normalised detection localization uncertainty (scaled between 0 and 1 for all detections within the cluster). μ is a constant which was set to $ln 0.1$. Calculation of topological features for a sampled cluster was performed using the GUDHI library as descried in Section 3.3. Any duplicated detections within a single sample were discarded for the purposes of calculating the persitence diagrams. The most common configuration is termed the consensus configuration and the agreement value, α, was defined as the the percentage of sampled clusters with the consensus configuration. This workflow is implemented within RSMLM.

Cluster measurements. Cluster area and volume were calculated using the convex hull of all detections in a cluster. Cluster density was defined as the number of cluster detections divided by the cluster area (volume in 3D). The number of holes per cluster was defined as the number of second order topological features in the corresponding persistence

diagram above a specified persistence threshold. Similarly, the number of voids corresponds to the number of third order features above a specified persistence threshold.

Radial averaging of clusters. Clusters were filtered for a specified topology, for example clusters with a single hole. Let $\mathbf{p} = \mathbf{p}_1,...,\mathbf{p}_n$ be the detection coordinates for a single filtered cluster, and $I = I_1,...,I_n$ be the corresponding detection intensities (photon counts). The intensity weighted center of mass, $\mathbf{C}$, is calculated as,

$$\mathbf{C} = \frac{\sum_{i=1}^{n} I_i \mathbf{p}_i}{\sum_{i=1}^{n} I_i} \tag{2}$$

For each detection, $\mathbf{p}_i$, the euclidean distance, $d(\mathbf{p}_i, \mathbf{C})$ to the corresponding centre of mass was then calculated. To produce the average radial distribution the distances $d(\mathbf{p}_i, \mathbf{C})$ across all filtered clusters were grouped into a single histogram, $H = H_1,..,H_j$. Each bin has a width of 10 nm and bin maxima r_j ranging from 10 to 150 nm. To normalise the distribution in 2D the value for each bin was divided by the corresponding bin area;

$$H'_j = \frac{H_j}{\pi(r_j^2 - r_{j-1}^2)} \tag{3}$$

In 3D the normalisation divides by the corresponding bin volume;

$$H'_j = \frac{H_j}{\frac{4}{3}\pi(r_j^3 - r_{j-1}^3)} \tag{4}$$

Finally the radial distribution was scaled between 0 and 1 ($H''_j = (H'_j - \min H')/(\max H' - \min H')$).

Simulation of data for comparison of clustering algorithms. Simulations for low, high and mixed (a mixture of high and low) density clusters were generated, either in close proximity or well separated. For each of these six scenarios four Gaussian clusters were placed on a field of view of size 400 nm^2 for well separated clusters, 300 nm^2 otherwise. The standard deviation for low and high density clusters was set to 5 nm and 20 nm respectively. For mixed density simulations two high density and two low density clusters were generated. For well separated simulations all clusters were separated by 200 nm. Otherwise low and high density clusters were separated by 100 nm and 60 nm respectively. After generating molecule locations the dSTORM imaging process was simulated. Simulations were repeated twenty times and algorithm performance, for a given parameter set, was averaged across all repeats.

Simulation of nano-structures for topological analysis. For ring simulations 40 molecules were even spaced on the circumference of a circle with radius 60 nm. For hollow sphere simulations 100 molecules were randomly placed on the surface of a sphere with radius 75 nm. The standard deviation for Gaussian clusters was set to the ring (or sphere) radius / 1.5 (87% of molecules will lie within the radius). For all scenarios five clusters were distributed on a 1 μm^2, or 1 μm^3, field of view according to a uniform random distribution, with re-selection if closer to another cluster than the diameter of the ring (or sphere). For randomly distributed simulations molecules were placed on the field of view according to complete spatial randomness. There where 200 and 500 molecules per field of view for the 2D and 3D simulations respectively. After generating molecule locations the dSTORM imaging process was simulated. Simulations were repeated twenty times for each scenario.

Platelet preparation and spreading. Human washed platelets were prepared from blood samples donated by healthy, consenting volunteers (local ethical review no: ERN-11-0175). Blood was drawn via venipuncture into sodium citrate as the anticoagulant and then acid/citrate/dextrose (ACD) added to 10% (v:v). Blood was centrifuged at 200 xg for 20 min. Platelet rich plasma (PRP) was removed and then centrifuged at 1000 xg for 10 min in the presence of 0.1 μ g/ml prostacyclin. Plasma was removed and the platelet pellet was resuspended in modified Tyrode's buffer (134 mM NaCl, 0.34 mM Na_2HPO_4, 2.9 mM KCl, 12 mM $NaHCO_3$, 20 mM HEPES, 5 mM glucose, 1 mM $MgCl_2$; pH 7.3) containing ACD and 0.1 μg/ml prostacyclin before being centrifuged for 10 min at 1000 xg. The washed platelet pellet was resuspended in modified Tyrode's buffer, left to rest for 30 min and the platelet count adjusted to 2×10^7 platelets/ml.

Platelet spreading and immunolocalisation. Glass-bottom MatTek dishes were coated with 10 μg/ml Horm collagen (diluted in manufacturer supplied diluent; Takeda, UK) overnight at 4 °C before being blocked in 5 mg/ml BSA for 1 hour at room temperature. Washed platelets were added to the dishes and allowed to spread for 45 min at 37 °C before the addition of the Syk inhibitor PRT060318 (10 μM) or DMSO control, each diluted in modified Tyrode's buffer. The platelets were returned to 37 °C for a further 15 min before being washed once in PBS and then fixed in 10% formalin solution for 10 min. Following PBS washes the platelets were permeabilised with 0.1% Triton X-100 for 5 min and then washed in

PBS and blocked for 30 min in block buffer (1% BSA, 2% goat serum in PBS). The platelet integrin $\alpha 2\beta 1$ was immuno-labelled with 5 μg/ml anti-CD49b (clone 16B4; AbD Serotec) diluted in block buffer for 1 hour at room temperature. Platelet integrin $\alpha 2\beta 1$ was secondary labelled with anti-mouse-Alexa647 and F-actin was labelled with phalloidin-Alexa488 (both Invitrogen and diluted 1:300 in block buffer). Platelets were washed and stored in PBS.

dSTORM imaging of platelet integrin $\alpha 2\beta 1$. Labelled platelets were imaged on a Nikon N-STORM system using a 100 $\times$ 1.49 NA TIRF objective. The system contains a Ti-E stand with Perfect Focus, a Andor IXON Ultra 897 EMCCD camera and a Agilent Ultra High Power Dual Output Laser bed (containing a 170 mW 647 nm laser and a 20 mW 405 nm laser). DIC and TIRF images of the platelets were taken to identify areas containing platelets and collagen fibres. To induce fluorophore blinking of the Alexa647 labelled integrin, platelets were imaged in a PBS-based buffer containing 100 mM MEA, 50 ug/ml glucose oxidase and 1 ug/ml catalase, pH 7.5, as detailed in (Metcalf *et al.*, 2013). For each image, 20,000 frames were captured using NIS Elements 4.2 with an exposure time of 20 ms, gain 300 and conversion gain 3. Detections were localised using the ThunderSTORM plugin for Fiji with a Gaussian PSF model and maximum likelihood fitting (Ovesnỳ *et al.*, 2014). Image visualisations were produced with the normalised Gaussian method.

Supplementary Note 2: A guide to parameter selection

Topological Mode Analysis Tool (ToMATo). Our implementation of ToMATo has two free parameters; firstly the search radius, r, which is used to calculate both the detection density estimate, f, and the linking graph, G, and secondly the persistence threshold, τ. First, consider r. If r is set too low then neighbouring detections will not be linked in G, and f will not be a useful estimate of detection density for the purposes of segmentation and clustering. This can be seen in the sharp cut-off in performance for very low values of r in **Supplementary Fig. 4**. If r is set too high then resolution is lost in G and f. The smallest structures will be smoothed over and ToMATo will not be able to segment them. In practice for nano-scale clustering we recommend starting with $r = 20$ nm and adjusting if appropriate (**Fig. 2**). To segment larger structures, or structures with a lower detection density, a larger r can be beneficial. For example to segment endocytic sites we used $r = 40$ nm (**Supplementary Fig. 15**) and to segment a whole cell we used $r = 70$ nm (**Supplementary Fig. 8**). Having set r the ToMATo diagrams should be computed and plotted. In simple cases these can be visually inspected to set a sensible persistence threshold, τ (**Fig. 1**). In more complex cases it may not be possible to choose τ based on visual inspection of the diagram (**Fig. 2**), however we always recommend plotting them. As the value of τ is increased more candidate clusters will fall below the threshold and will either be merged to a stronger candidate or be considered noise. Therefore if we are trying to segment larger structures with in-homogeneous detection density then a larger value for τ is appropriate, for example we used $\tau = 20$ detections to segment endocytic sites (**Supplementary Fig. 15**). If we are looking at nano-scale clustering and would like to segment clusters which are close together then τ should be relatively low. For example we used $\tau = 10$ detections to cluster receptors binding to collagen fibres (**Fig. 2**). Alternatively if we know how many structures, n, are in a dataset then we can select τ automatically by selecting the n candidates with the highest persistence (**Supplementary Fig. 8**).

Persistent homology. For topological analysis the key free parameter is the persistence threshold, T. **Supplementary Fig. 12** demonstrates the effect of varying T for simulated data. To summarise, if T is set very low then we will detect lots of noisy features. It is clear that this has happened with a sub-sampling approach as the consensus results have low agreement. This means we have low confidence in the results. If T is set very high we lose resolution and cannot detect smaller, less stable features. Small changes in intermediate values have little effect on the end result. All 2D examples in this paper use $T = 15$ nm (**Fig. 3, 4**).

References

Andronov, L., Orlov, I., Lutz, Y., Vonesch, J.-L., and Klaholz, B. P. (2016). ClusterViSu, a method for clustering of protein complexes by Voronoi tessellation in super-resolution microscopy. *Scientific reports*, **6**, 24084.

Chazal, F., Guibas, L. J., Oudot, S. Y., and Skraba, P. (2013). Persistence-based clustering in Riemannian manifolds. *Journal of the ACM (JACM)*, **60**(6), 41.

Ester, M., Kriegel, H.-P., Sander, J., Xu, X., *et al.* (1996). A density-based algorithm for discovering clusters in large spatial databases with noise. In *Kdd*, volume 96, pages 226–231.

Levet, F., Hosy, E., Kechkar, A., Butler, C., Beghin, A., Choquet, D., and Sibarita, J.-B. (2015). SR-Tesseler: a method to segment and quantify localization-based super-resolution microscopy data. *Nature methods*, **12**(11), 1065.

Metcalf, D. J., Edwards, R., Kumarswami, N., and Knight, A. E. (2013). Test samples for optimizing storm super-resolution microscopy. *Journal of visualized experiments: JoVE*, (79).

Ovesnỳ, M., Křížek, P., Borkovec, J., Švindrych, Z., and Hagen, G. M. (2014). ThunderSTORM: a comprehensive ImageJ plug-in for PALM and STORM data analysis and super-resolution imaging. *Bioinformatics*, **30**(16), 2389–2390.

Owen, D. M., Rentero, C., Rossy, J., Magenau, A., Williamson, D., Rodriguez, M., and Gaus, K. (2010). PALM imaging and cluster analysis of protein heterogeneity at the cell surface. *Journal of biophotonics*, **3**(7), 446–454.

Rubin-Delanchy, P., Burn, G. L., Griffié, J., Williamson, D. J., Heard, N. A., Cope, A. P., and Owen, D. M. (2015). Bayesian cluster identification in single-molecule localization microscopy data. *Nature methods*, **12**(11), 1072.

Sage, D., Kirshner, H., Pengo, T., Stuurman, N., Min, J., Manley, S., and Unser, M. (2015). Quantitative evaluation of software packages for single-molecule localization microscopy. *Nature methods*, **12**(8), 717.

Supplementary Note 3: Tables

Simulation	Total error (%)	Error breakdown (%)		
		False positive	False negative	Wrong cluster
**Low**	13.0	46.0	6.6	47.4
**Mixed**	10.7	39.5	15.5	45.1
**High**	9.7	35.6	9.1	55.3
**Low Sep.**	6.1	77.2	22.4	0.4
**Mixed Sep.**	5.1	79.9	20.1	0.0
**High Sep.**	3.7	76.4	23.6	0.0

Table S1. Error classification for ToMATo clustering across six different simulation scenarios; low, mixed and high density clusters either in close proximity, or well separated (Sep.). Total error is the percentage of incorrectly assigned detections. Simulations were repeated twenty times and the mean results are shown for the parameter set which produced the lowest total error. Errors were split into three categories; noise detections which were assigned to clusters (false positives), clusters detections which were designated noise (false negatives) and clusters detections which were assigned to the wrong cluster. For the scenarios where clusters are well separated the dominant source of errors are false positives. For the scenarios where clusters are close together incorrect cluster assignments are the most prominent source of error.

	ToMATo	DBSCAN	Ripley K	Voronoi, 1st	Voronoi, Zero
**Processing Time (s)**	9 (RSMLM)	33 (RSMLM)	50 (RSMLM)	35 (SR-Tesseler)	-

Table S2. Computation time for different clustering methods for a representative dSTORM acquisition from the integrin $\alpha 2\beta 1$ dataset (DMSO). The file contained 1.04×10^6 detections after filtering (2.02×10^6 detections raw). The filtered detection list (full field of view) was clustered using each algorithm and the computational time was recorded. RSMLM was used for ToMATo, DBSCAN and Ripley's K based clustering. The Voronoï based methods implemented in RSMLM were not able to process a dataset of this size so SR-Tesseler (version 1.0.0.1) was used (first order density estimate) (Levet *et al.*, 2015). For ToMATo, DBSCAN and Ripley's K based clustering the search radius was set to 20 nm. The local density thresholds for ToMATo, DBSCAN, Ripley's K based, and Voronoï diagram (first order density estimate) based clustering were set to 10 detections, 15 detections, 15$(L-r)$ and 2.5 (normalised) respectively. Computation was performed on a desktop computer (Windows 10, 64-bit) with 128GB RAM, a Intel Core i7-6900K CPU and a NVIDIA Quadro P4000 graphics card. This comparison is intended to provide an estimate of computation time using the tested tools for a substantial dataset and is not a comparison of algorithmic complexity, or theoretical speed.

	ToMATo	PH, full data	PH, clustered	PH, sub-sampled
**Processing Time (s)**	2	887	88	2026

Table S3. Computation time for ToMATo clustering, persistent homology analysis run on the full dataset (PH, full data), persistent homology analysis run on each cluster sequentially (PH clustered) and persistent homology analysis run on each cluster sequentially with the sub-sampled consensus approach (PH, sub-sampled). An example dSTORM dataset with labelled nuclear pore component Nup107 was used for test purposes. The file contained 2.10×10^5 detections after filtering (9.75×10^5 detections raw). Note that processing each cluster sequentially reduced the computation time by approximatelly a factor of ten for this dataset. The sub-sampling approach takes substantially longer (34 minutes for 50 sampling iterations). However as the approach is fully automated and batch compatible this is not limiting for most applications. Computation was performed on a desktop computer (Windows 10, 64-bit) with 128GB RAM, a Intel Core i7-6900K CPU and a NVIDIA Quadro P4000 graphics card. This comparison is intended to provide an estimate of computation time for a substantial dataset and is not a comparison of algorithmic complexity, or theoretical speed.

Supplementary Note 4: Videos

Video S1. Illustrative example showing the filtration process used to produce persistence diagrams. The detections are spaced evenly on the circumference of two circles. Circles of increasing radius are placed on top of detections (left panel). When two circles overlap a line is added to the Rips complex, when three circles overlap a triangle is added. When a hole is formed in the Rips complex its birth scale is added to the diagram as a red point. When the hole dies its final position in the persistence diagram is recorded as black point. The dotted line shows the persistence threshold.

Video S2. Filtration example for 2D dSTORM simulations of Gaussian clusters and rings. The dotted line shows the persistence threshold.

Video S3. Filtration example for 3D simulations of Gaussian clusters and spheres. There are persistence diagrams to record both holes and voids. Dotted lines show the persistence thresholds.

Supplementary Note 5: Figures

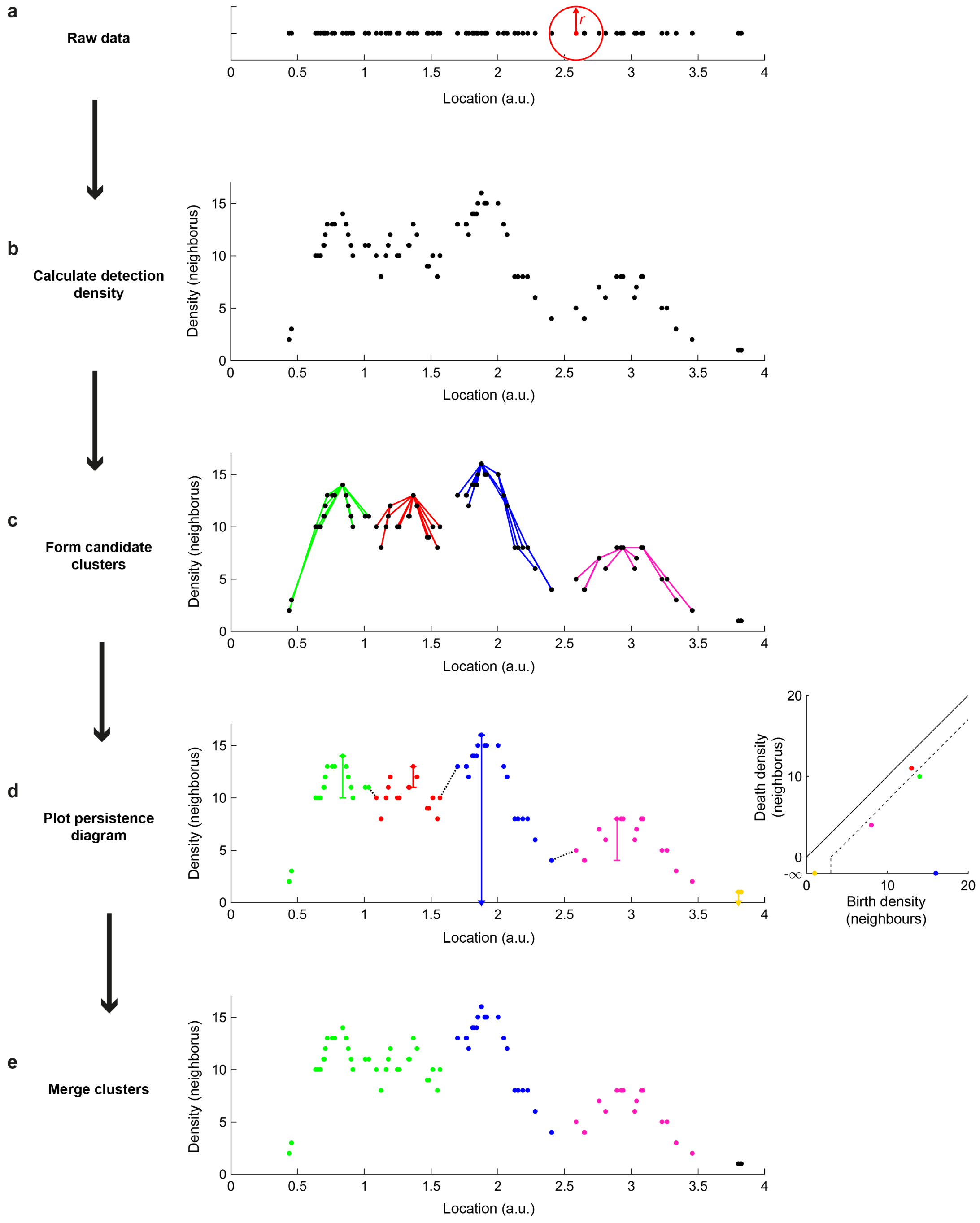

Fig. S1. Illustrative example to demonstrate the ToMATo algorithm. (**a**) 1D data was simulated for three Guassian clusters with unequal density. The first step is the estimation of detection density. This is done by calculating the number of neighbouring detections within a fixed search radius, r. (**b**) Plot showing the estimated density for each detection. (**c**) Next a graph is formed which links all detections within distance, r. Candidate clusters are formed by following the density gradient within this graph until a local maximum is reached. This is called mode seeking, or hill climbing. The lines represent the density gradient and each candidate cluster is shown as a different colour. (**d**) For each candidate cluster the maximum density, known as the birth density, is recorded. We also find the death density, the saddle point at which the cluster merges to a stronger neighbour. The coloured solid lines represent the persistence; the difference between the birth and death densities (left). The right side shows the persistence diagram where for each candidate cluster the birth : death position is recorded. At this point a persistence threshold is chosen. Candidates below the threshold will be merged to neighboring candidates with persistence greater than, or equal to, the threshold. Here a value is chosen which selects the three most prominent clusters (dotted line). (**e**) Final result after cluster merging based on persistence. Only the three most prominent clusters remain. Detections which cannot be linked to a cluster above the persistence threshold are said be noise and are shown in black.

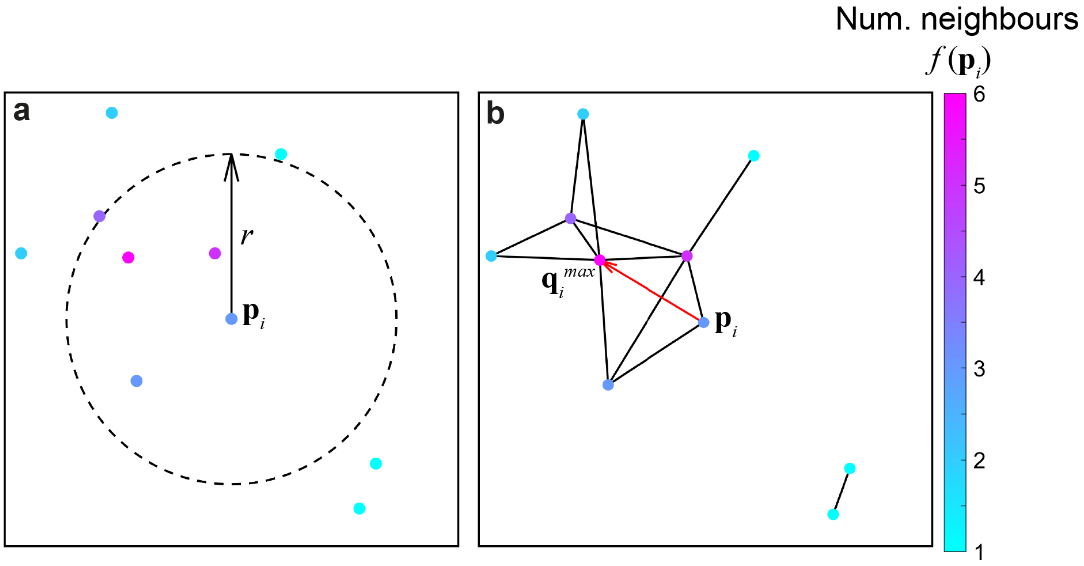

Fig. S2. Illustrative example to demonstrate the formation of the Rips graph and mode seeking for the ToMATo approach. (**a**) The density estimate, $f(\mathbf{p}_i)$, is calculated by counting the number of detections within a specified search radius, r. The search region is shown for a specific detection, $\mathbf{p}_i$. Detections are coloured based on $f(\mathbf{p}_i)$. (**b**) The Rips graph, $G(\mathbf{p})$, is formed by linking all detection separated by at most r (black lines). Candidate clusters are then formed by linking each detection, $\mathbf{p}_i$, to the neighbour, $\mathbf{q}_i^{\max}$, which has higher density than both $\mathbf{p}_i$ and all other neighbouring detections (red line).

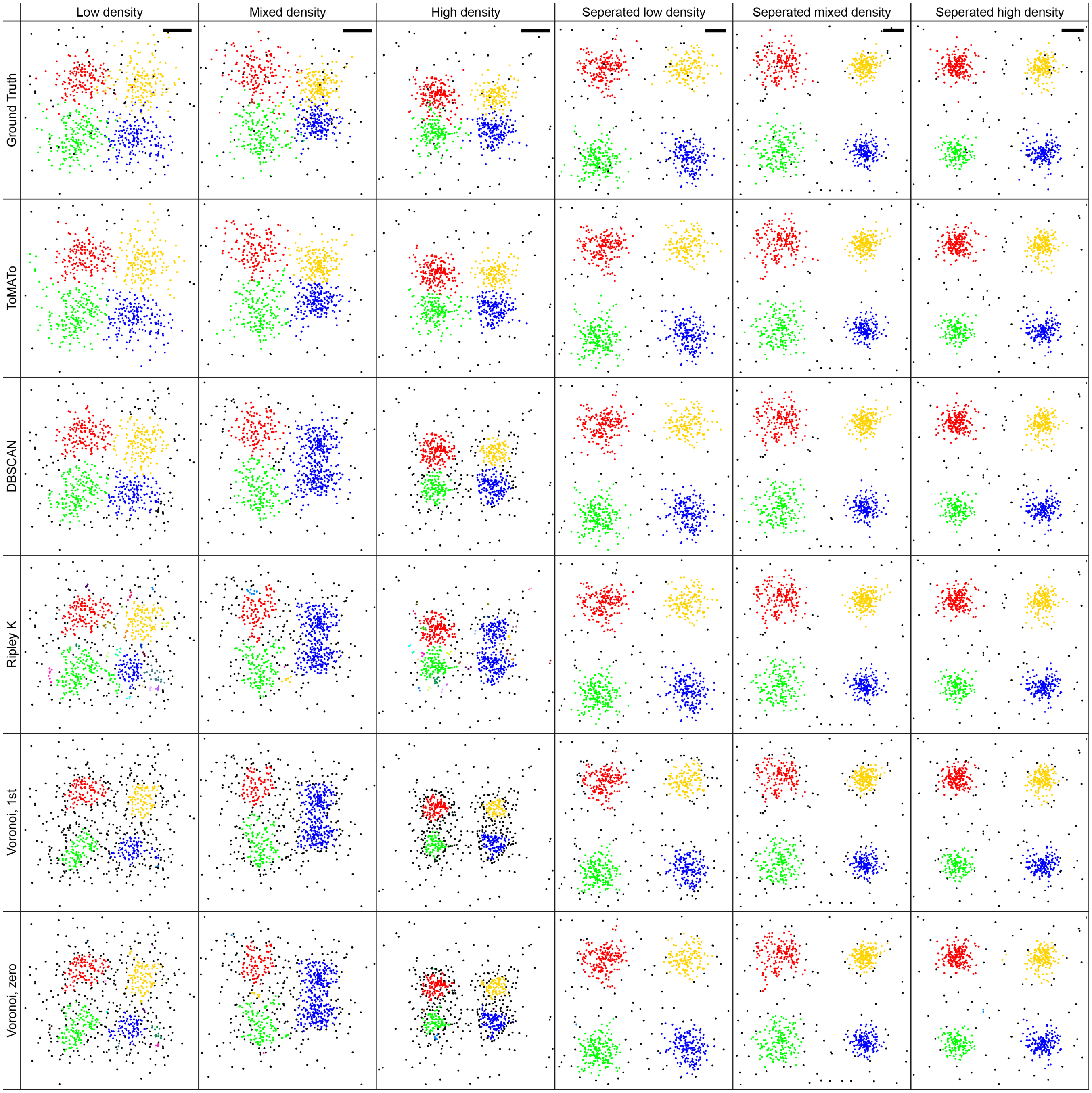

Fig. S3. Example cluster results. For each algorithm and scenario the parameters which produced the highest mean performance were used. Scale-bar 50 nm.

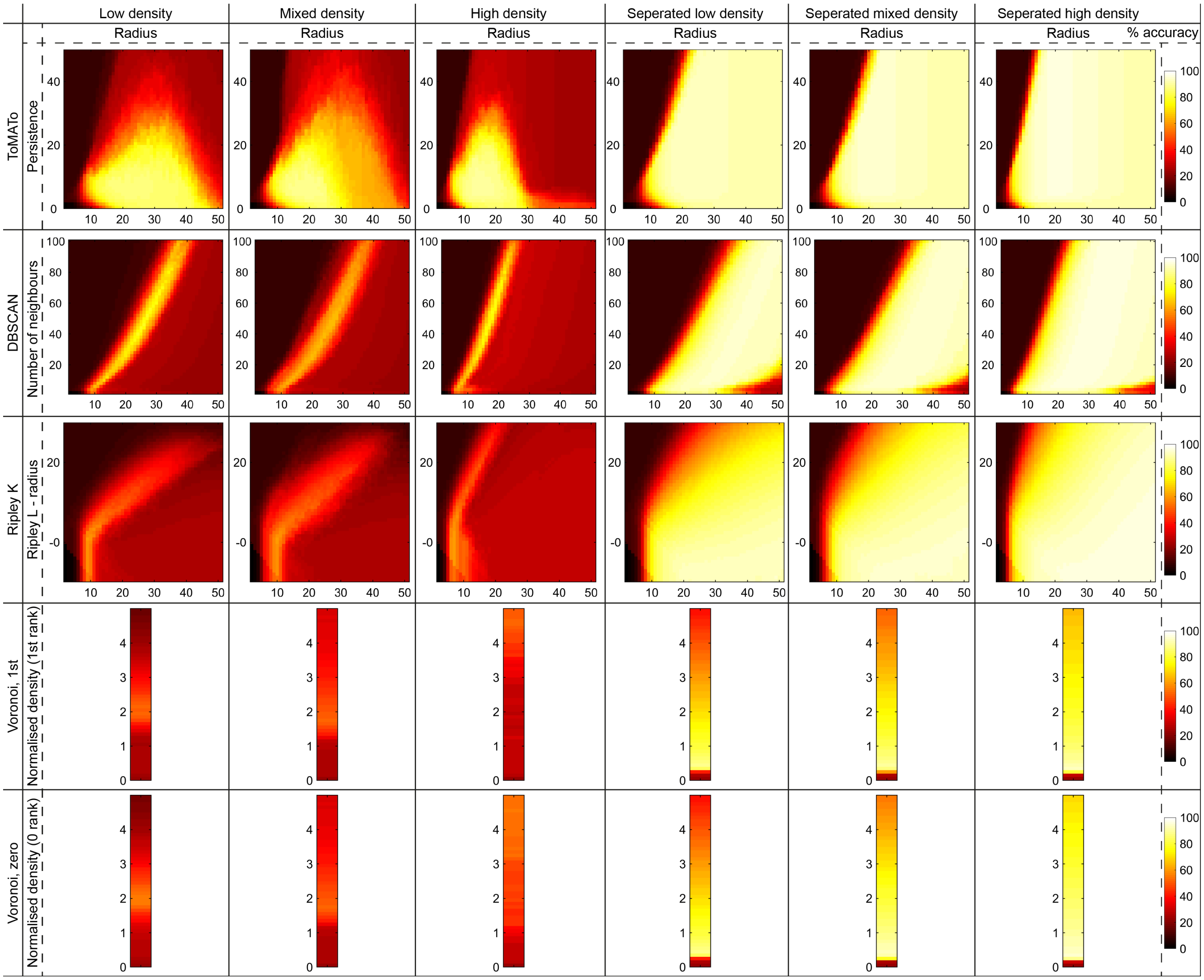

Fig. S4. Heatmaps showing the performance of each clustering algorithm across all clustering scenarios and parameter sets. Performance is defined as the percentage of correctly assigned detections and was averaged across twenty simulations.

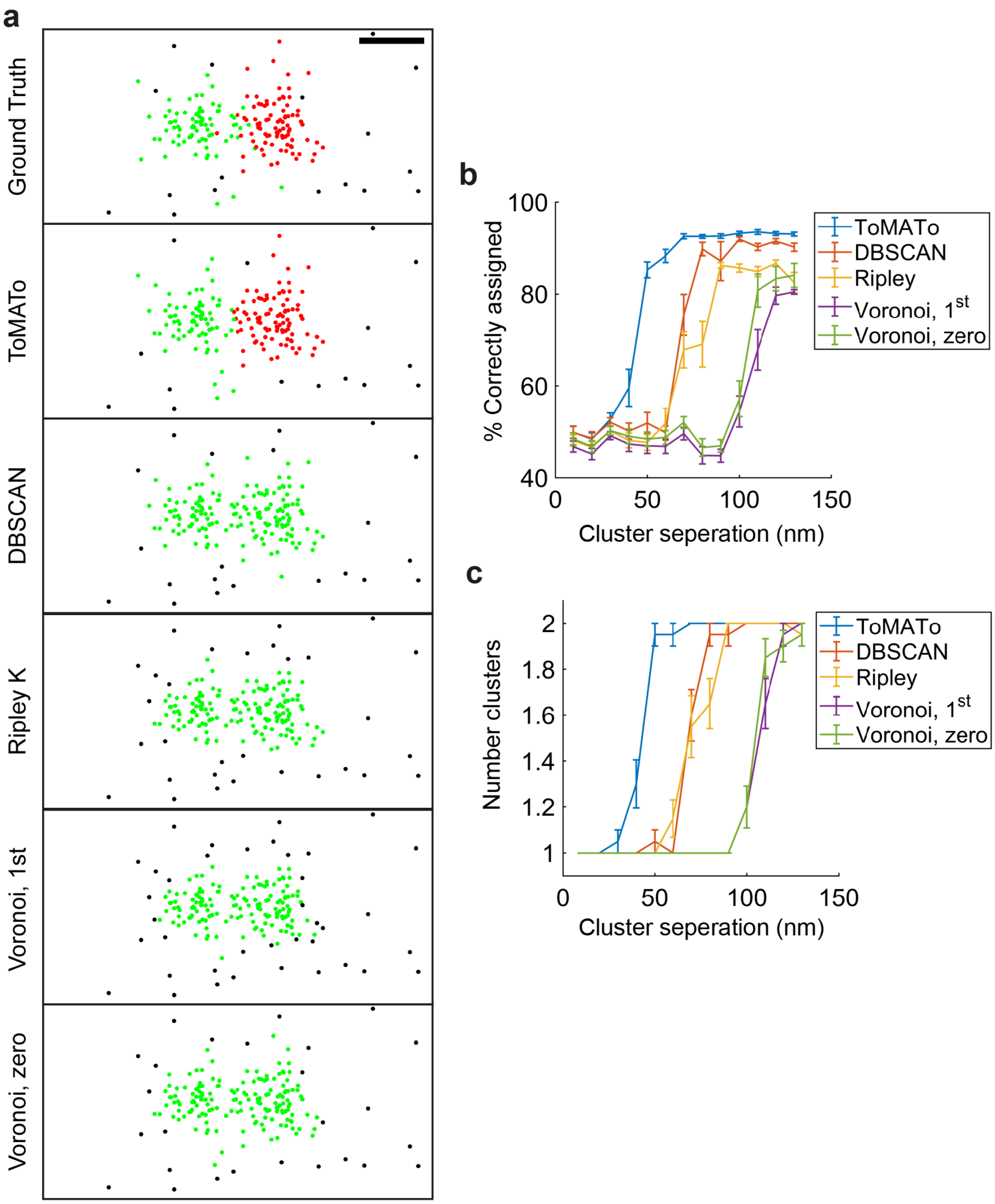

Fig. S5. Evaluation of clustering performance when varying cluster separation in a fixed parameter setting. 2D dSTORM simulations of two Gaussian clusters, each containing 20 molecules and with a standard deviation of zero, were generated. The cluster separation was varied from 10 to 130 nm and the field of view size was 300 $\times$ 150 nm. All other simulation parameters are as described in Section 3.4 and the supplementary methods. (**a**) Example ground truth simulation and clustering results for a cluster separation of 60 nm. Scale-bar 50 nm. (**b**) Performance was defined as the percentage of correctly assigned detections and was averaged across twenty simulations. For each tested algorithm the optimal fixed parameter set which produced the highest mean performance across all cluster separations was chosen. ToMATo outperforms other tested algorithms across all cluster separations with a large performance increase ($> 15\%$) between 50 and 70 nm, and a maximum performance increase of 36$\%$ at 60 nm. Error bars are the standard error of the mean. (**c**) Mean number of clusters detected for the parameter set which produced the highest mean performance. Only ToMATo is able to consistently segment both clusters in the 50 to 70 nm range. Error bars are the standard error of the mean.

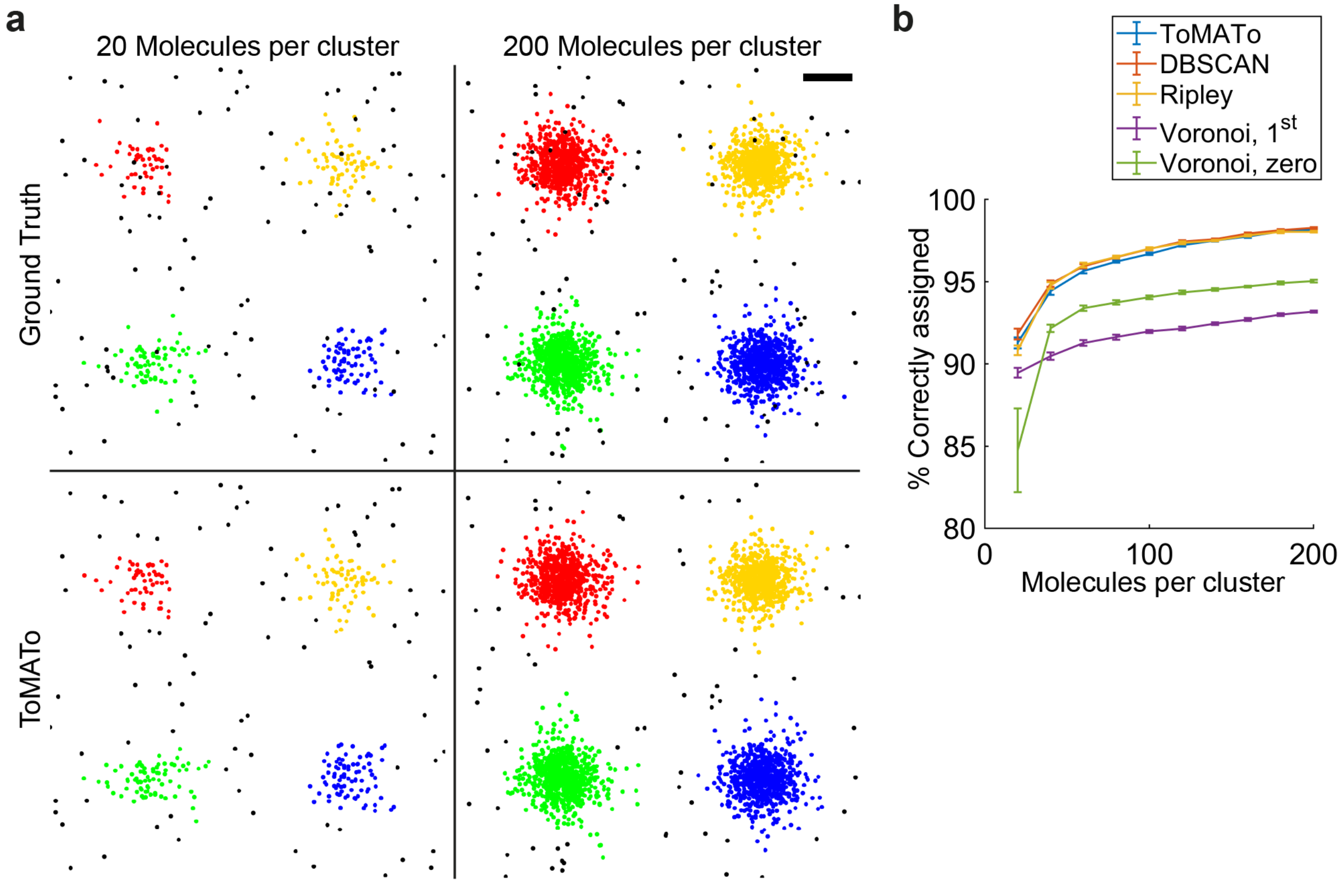

Fig. S6. Evaluation of clustering performance for varying cluster density in a fixed parameter setting. (**a**) 2D dSTORM simulations of four well separated Gaussian clusters were generated where the number of molecules per cluster was varied from 20 to 200. Scale-bar 50 nm. (**b**) Performance was defined as the percentage of correctly assigned detections and was averaged across twenty simulations. For each tested algorithm the optimal fixed parameter set which produced the highest mean performance across all cluster densities was chosen. ToMATo clustering is able to perform as well as any other tested algorithm for these simulations but does not offer a performance increase over DBSCAN and Ripley's K based clustering as the clusters are well separated. Error bars are the standard error of the mean.

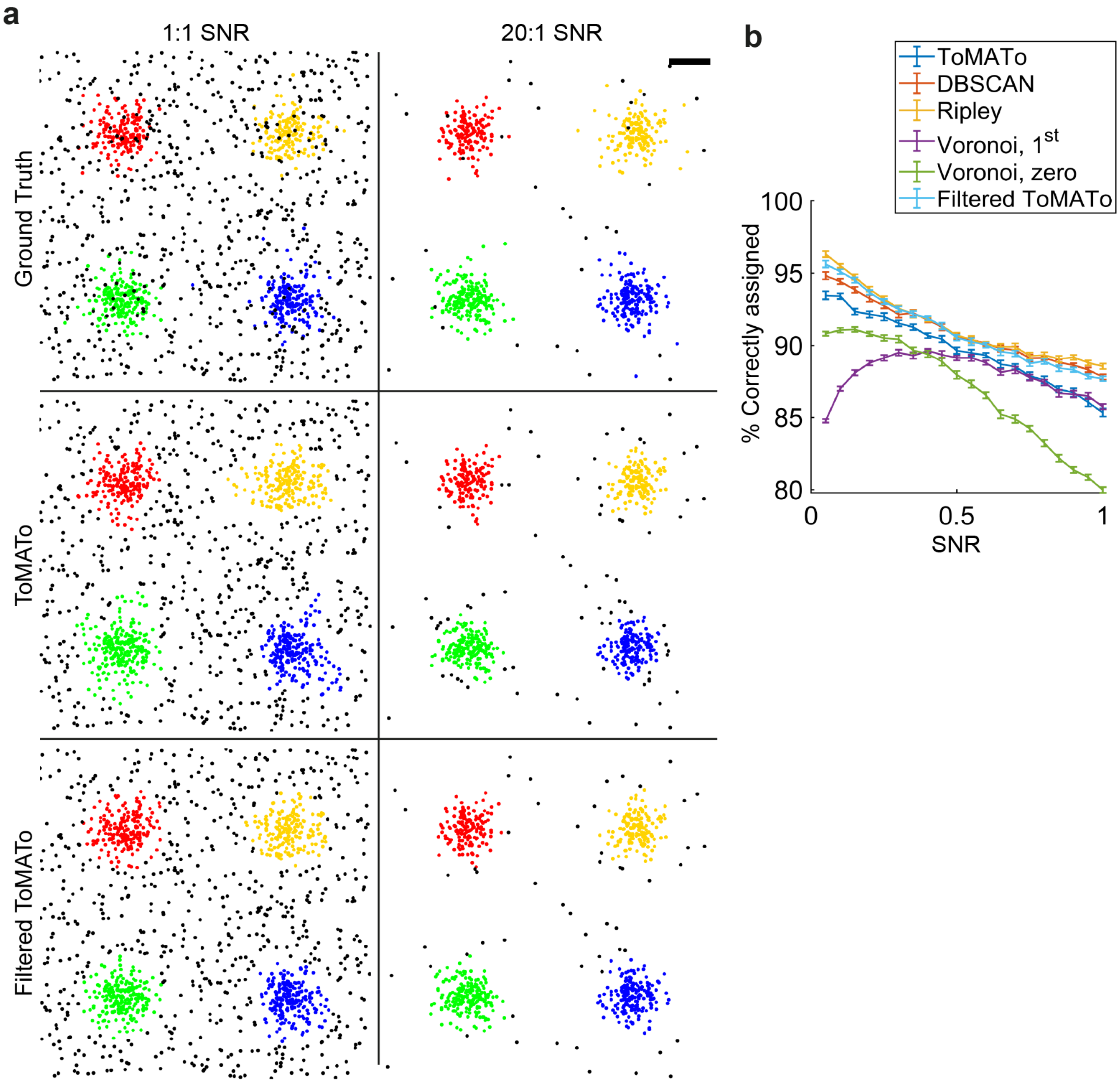

Fig. S7. Evaluation of clustering performance for varying noise levels in a fixed parameter setting. (**a**) 2D dSTORM simulations of four well separated Gaussian clusters each with fifty molecules were generated. The signal to noise ratio (SNR) was varied from 1:1 to 20:1. Results of the ToMATo clustering algorithm with, and without, automated density based pre-filtering are shown. Density pre-filtering was performed by removing all detections with a Ripley L value less than the search radius. This simply removes all detections with a density less than what would be expected from a random distribution. The search radius for the Ripley L calculations was chosen to be the same as the radius used in the ToMATo clustering algorithm. Scale-bar 50 nm. (**b**) Performance was defined as the percentage of correctly assigned detections and was averaged across twenty simulations. For each tested algorithm the optimal fixed parameter set which produced the highest mean performance across all SNR levels was chosen. Ripley's K based clustering had the highest overall performance for these simulations. We speculate this is because the Ripley K function is normalised to the number of detections in the field of view. Although the Voronoï based methods are also normalised for our simulations they were lowest performing. Note that performance of ToMATo is increased in these simulations by density based pre-filtering. Error bars are the standard error of the mean.

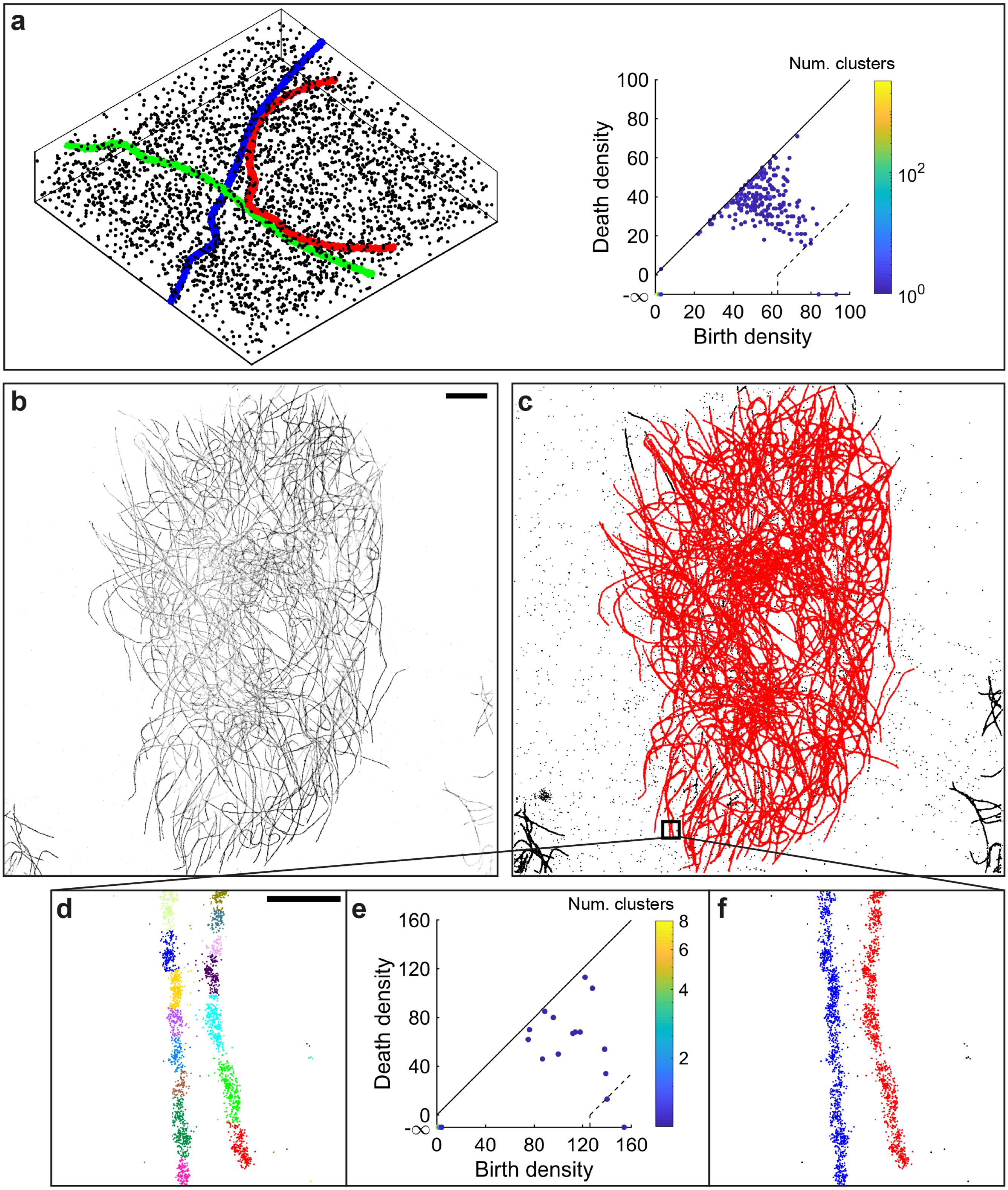
a
Num. clusters
Death density
Birth density
100
80
60
40
20
0
-∞
0
20
40
60
80
100
10^2
10^0
b
c
d
e
Num. clusters
160
120
80
40
0
-∞
0
40
80
120
160
8
6
4
2
Death density
Birth density
f

Fig. S8

Fig. S8 ***(previous page).*** Segmentation of tubular structures and whole cells using ToMATo. (**a**) Simulated 3D data of microtubules produced for the SMLM software challenge (Sage *et al.* (2015)). The ground truth molecule localizations were run through our dSTORM simulation engine. Segmentation result and ToMATo diagram shown. Density estimates were produced using a search radius of 30 nm. The persistence threshold was set to select the three most prominent clusters in the diagram. This demonstrates that ToMATo is capable of segmentation non-circular structures in 3D, even when they are close together. (**b**) Rendered dSTORM image of whole A549 cell labelled for α-tubulin. Scale-bar 5 μm. (**c**) Whole cell segmentation using ToMATo. Here we are unable to segment individual microtubules without a specialized method as the degree of overlap is too high. However by choosing the most prominent cluster in the persistence diagram the whole cell can be segmented. Density estimates were produced using a search radius of 70 nm. (**d**) Density modes for cropped region. Scale-bar 0.5 μm. (**e**) Persistence diagram for cropped region. As the two microtubules are non-overlapping they can be separated by choosing a persistence threshold which selects the two most prominent clusters. (**f**) Clustering result after merging based on persistence.

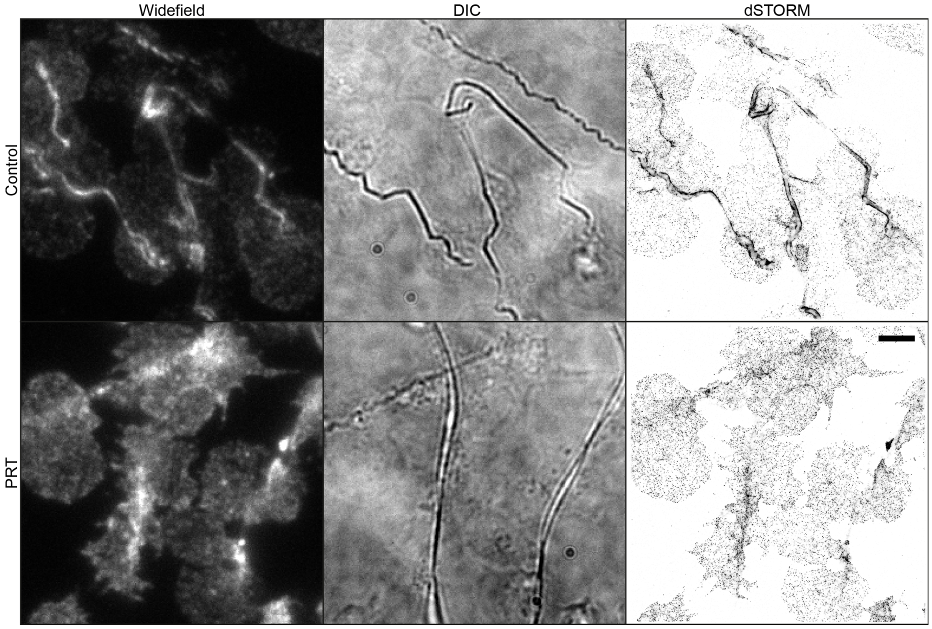

Fig. S9. Imaging of integrin $\alpha 2\beta 1$ by dSTORM. Platelets were seeded on collagen fibers and treated either with PRT060318, or DMSO (control). Representative widefield, DIC and dSTORM images are shown. Collagen fibers can be seen in the DIC image. Scale-bar 5 μm.

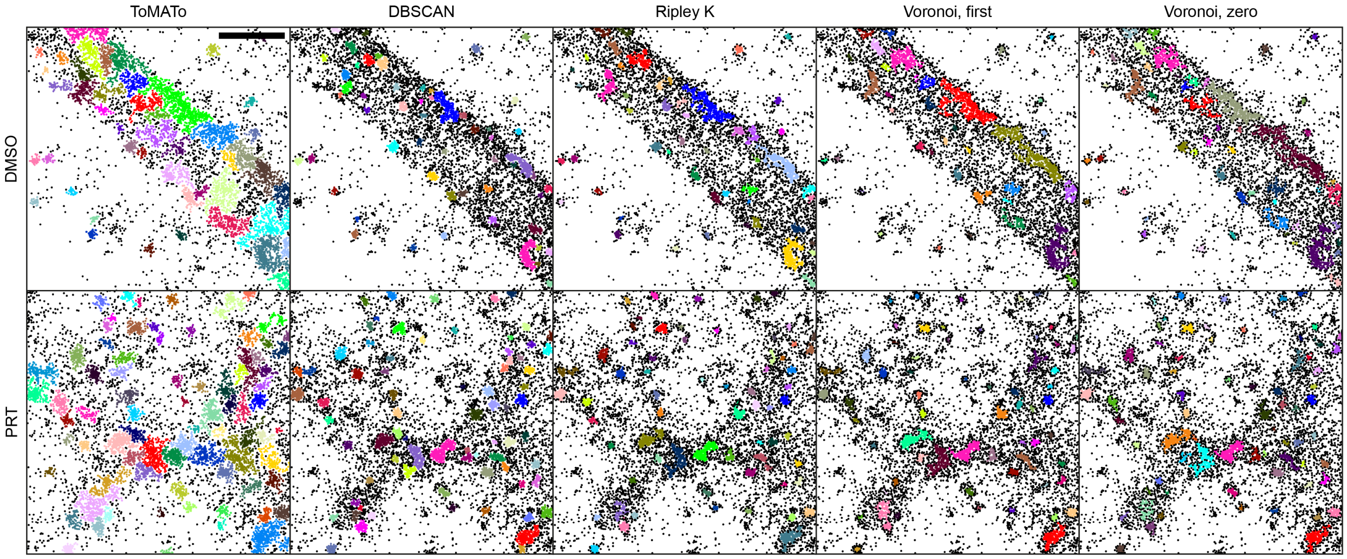

Fig. S10. Comparison of methods for segmentation of integrin $\alpha 2\beta 1$ clusters in platelets seeded on collagen fibers, treated either with PRT060318, or DMSO (control). Images show representative cropped regions. Parameters were selected based on visual evaluation of the segmentation for representative images across conditions and biological replications. Visual inspection implies ToMATo is able to segment clusters which are close together without trimming the edges of the clusters. For ToMATo, DBSCAN and Ripley's K based clustering the search radius was set to 20 nm. The local density thresholds for ToMATo, DBSCAN, Ripley's K based, Voronoï diagram (first order density estimate) and Voronoï diagram (zero order density estimate) based clustering were set to 10 detections, 15 detections, 15($L - r$), 2.5 (normalised) and 2.5 (normalised) respectively. Scale-bar 500 nm.

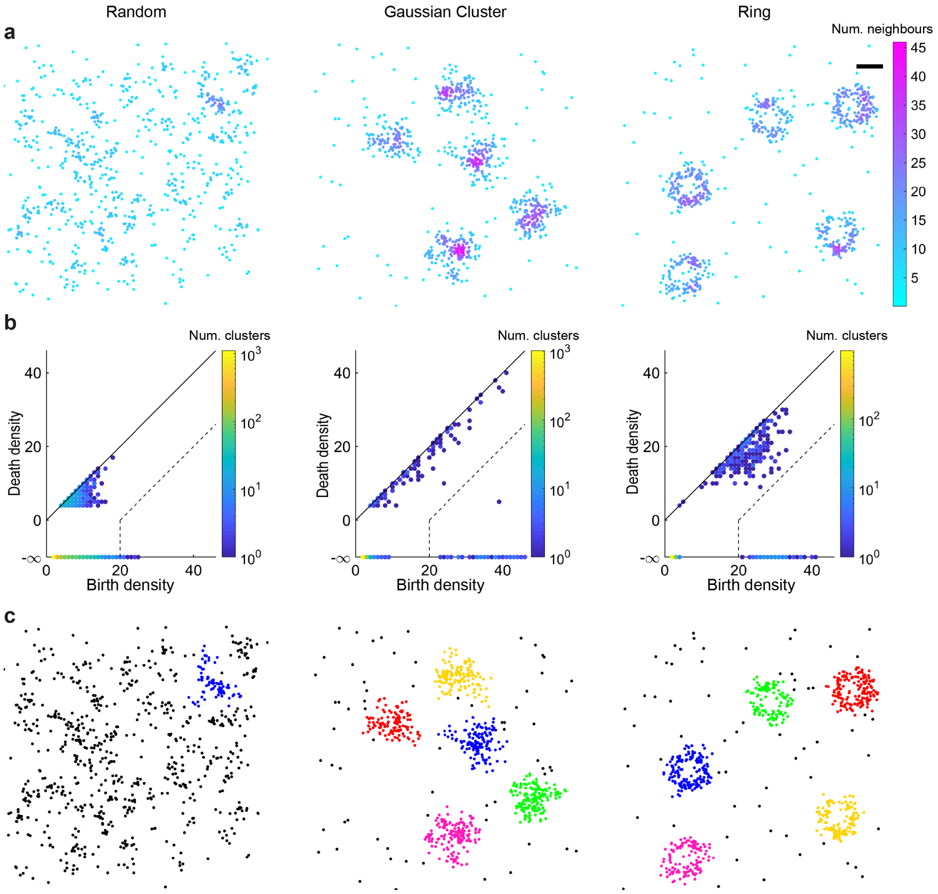

Fig. S11. Persistence based clustering as a pre-processing step for topological analysis. (**a**) Detection density for simulations of randomly distributed molecules, Gaussian clusters and rings were estimated by counting the number of other detections within 30 nm. Scale-bar 100 nm. (**b**) ToMATo diagrams showing the birth and death coordinates for all candidate clusters. For unbiased visualisation clusters across repeated simulations have been grouped. A persistence threshold of 20 nm was used to merge clusters (dotted line). (**c**) Final clustering results after merging. Noise detections are shown in black.

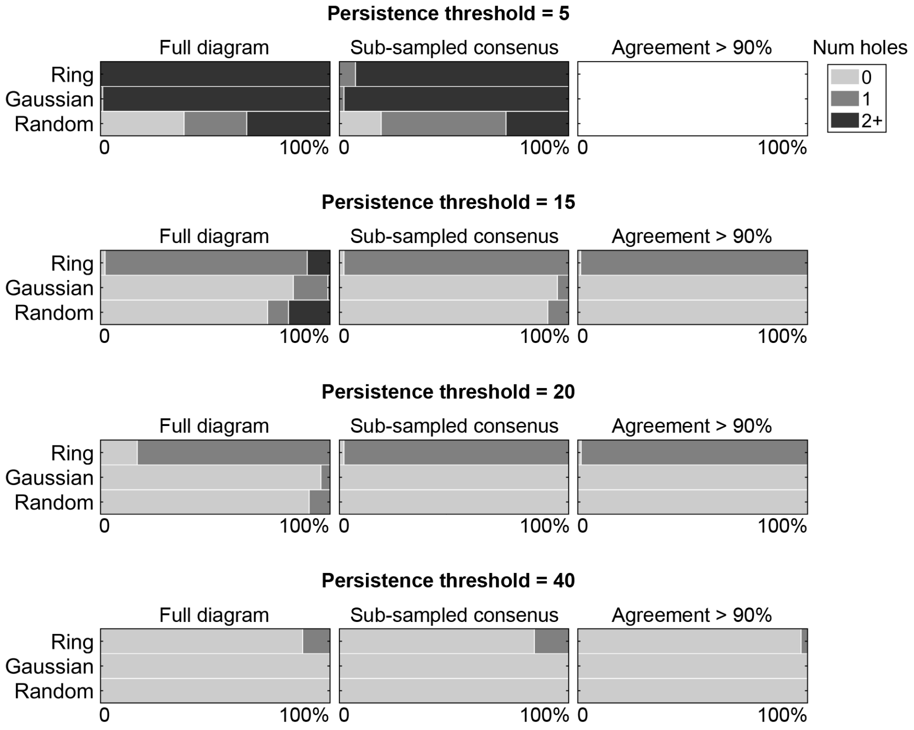

Fig. S12. Simulations for randomly distributed molecules, Gaussian clusters and rings with 60 nm radius were first segmented using ToMATo and then processed using persistent homology. The plots show the effect of varying persistent threshold, T, on the percentage of clusters in different topological configurations. For each scenario and threshold the cluster configurations are calculated using either the full diagram, the sub sampled consensus or the sub-sampled consensus with agreement, $\alpha > 90\%$. When the persistence threshold is set very low ($T = 5$) then most clusters have two or more holes. Most of these holes are very unstable and are not significant. This is clear as no clusters for $T = 5$ have $\alpha > 90\%$. When the persistence threshold is set at intermediate values ($T = 15$ or $T = 20$), then we find that the majority of clusters are assigned the correct classification (one hole for ring, zero for Gaussian and zero for random clusters). The rate of classification errors are reduced with a sub-sampling approach and reduced further by filtering for $\alpha > 90\%$. The conclusions drawn from the data would be the same for $T = 15$ and $T = 20$. For very high persistent thresholds ($T = 40$) most holes in the ring simulations are not detected. This is because T sets a bound on the sensitivity of the method, too high and we can only detect the most robust features. To set T we recommend inspecting the persistence diagram and selecting an intermediate value. It is advisable to run the analysis with several values of T to evaluate variation in the results.

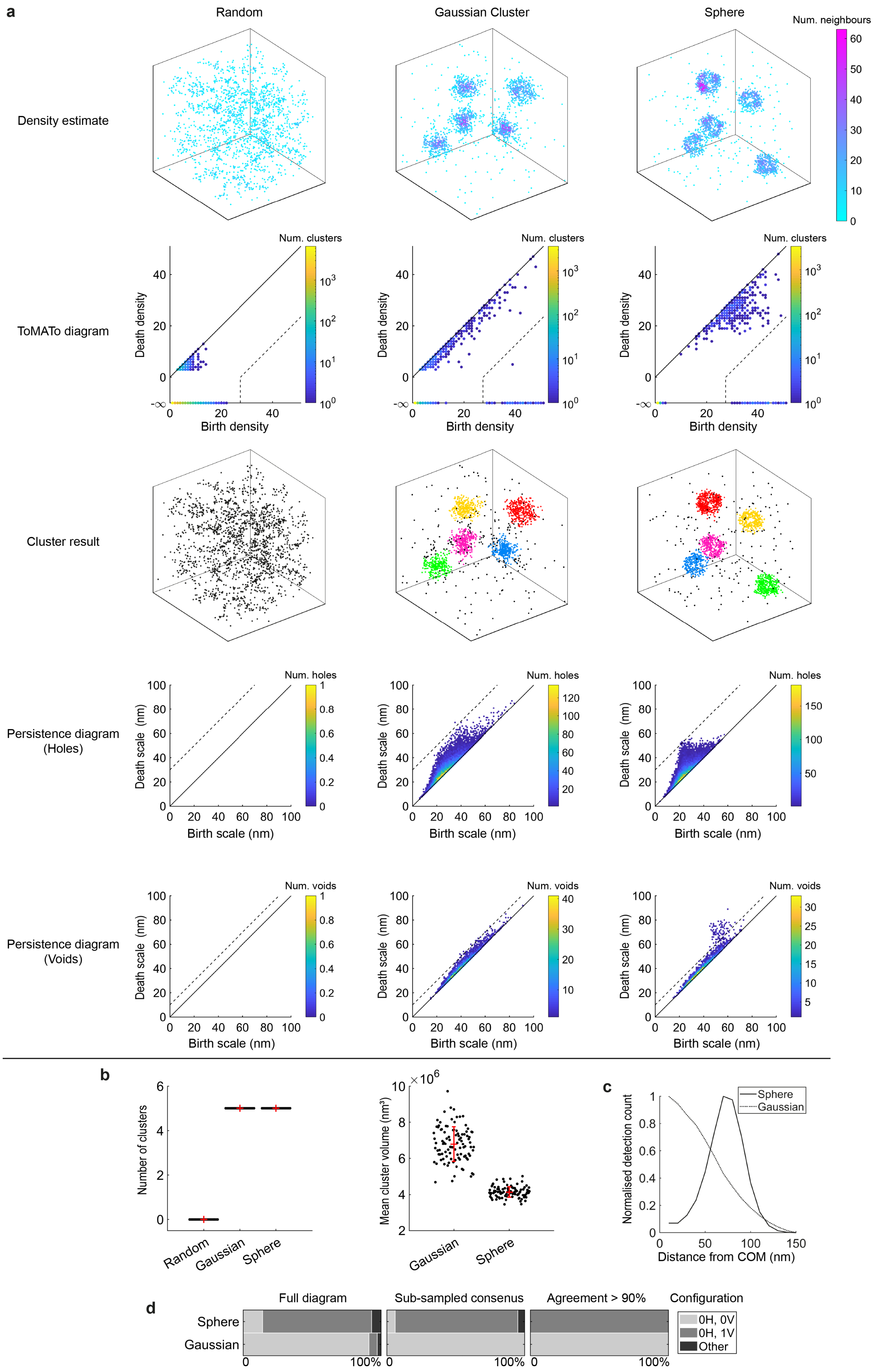
a
Random
Gaussian Cluster
Sphere
Num. neighbours
Density estimate
ToMATo diagram
Num. clusters
Death density
Birth density
Cluster result
Persistence diagram (Holes)
Num. holes
Death scale (nm)
Birth scale (nm)
Persistence diagram (Voids)
Num. voids
b
Number of clusters
Random
Gaussian
Sphere
Mean cluster volume (nm³)
c
Normalised detection count
Distance from COM (nm)
Sphere
Gaussian
d
Full diagram
Sub-sampled consensus
Agreement > 90%
Configuration
0H, 0V
0H, 1V
Other

Fig. S13

Fig. S13 *(previous page)*. Persistence based clustering and persistent homology for analysis of 3D SMLM datasets. (**a**) Example simulations for randomly distributed molecules, Gaussian clusters and hollow spheres with radius 75 nm. Detection density was estimated by counting the number of other detections within 40 nm. ToMATo diagrams were used to select a persistence threshold for merging of density clusters (27.5 detections, dotted line). After cluster merging persistent homology was performed to produce persistence diagrams for both 2D (holes) and 3D (enclosed voids) features. Features from all clusters were grouped into a single diagram per scenario and dimension. Persistence thresholds of 30 nm and 10 nm were selected for holes and voids respectively. (**b**) Number of clusters and mean cluster area. Error bars are mean $\pm$ s.d.. (**c**) Averaged radial distribution for clusters with agreement $> 90\%$. Peak of the profile for the spherical simulation lies at 80 nm. (**d**) Percentage of clusters with specified configuration for each scenario. This was calculated using either the full diagram, the sub-sampled consensus, or the sub-sampled consensus with agreement $> 90\%$. Clusters with a single void and no holes are dominant for the hollow sphere simulation. This is consistent with the topology of the simulated structures.

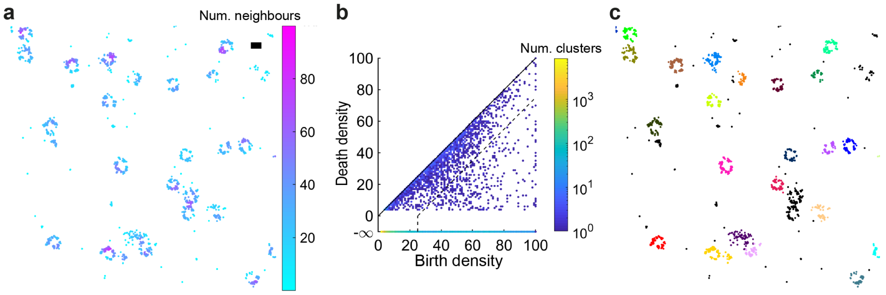

Fig. S14. Persistence based clustering of nuclear pore component Nup107 in 2D. (**a**) Density estimate was calculated using a search radius of 25 nm. Representative cropped field of view shown. Scale-bar 100 nm. (**b**) ToMATo diagram showing all candidate clusters. Persistence threshold set to 25 detections (dotted line). (**c**) Result of ToMATo clustering to segment nuclear pore complexes. Clusters were filtered by number of detections $(20 - 400)$ and area ($\pi \times 40^2 - \pi \times 100^2$nm^2).

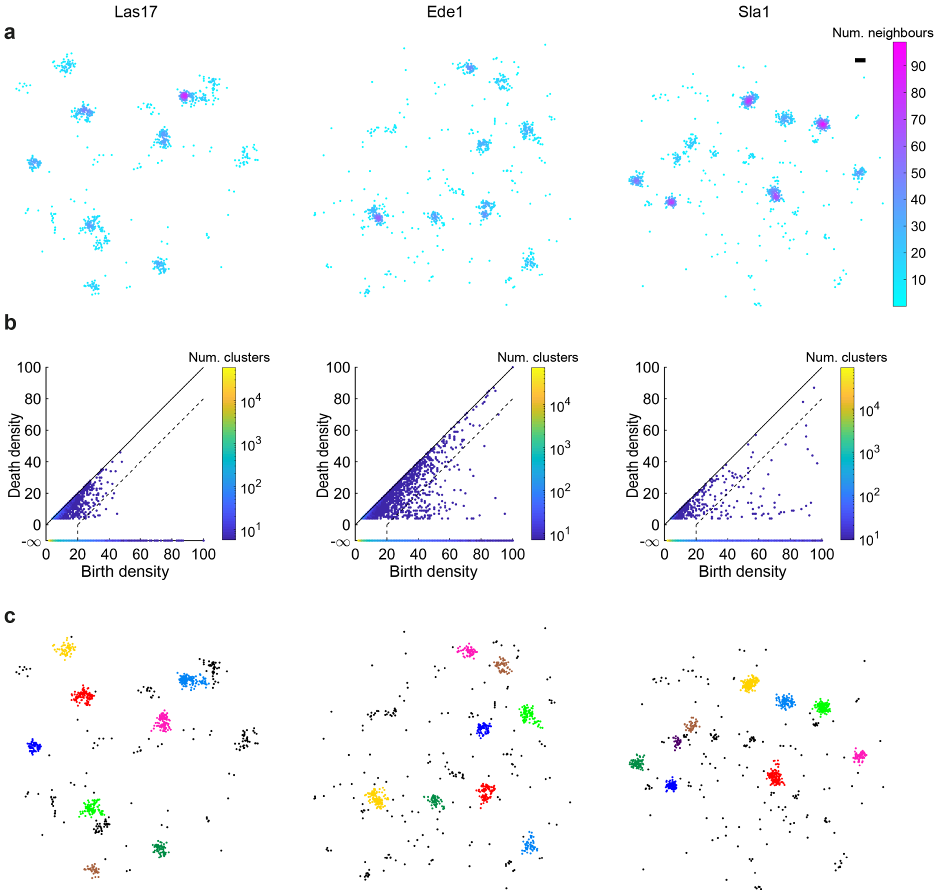

Fig. S15. Persistence based clustering of endocytic proteins; Las17, Ede1 and Sla1. (**a**) Density estimates were calculated using a search radius of 40 nm. Representative cropped field of view shown. Scale-bar 100 nm. (**b**) ToMATo diagrams showing candidate clusters. Persistence threshold set to 20 detections (dotted lines). All fields of view grouped into a single diagram per condition. (**c**) Result of ToMATo clustering to segment endocytic sites. Clusters were filtered by number of detections $(20+)$ and area $(\pi \times 30^2 - \pi \times 130^2\text{nm}^2)$.

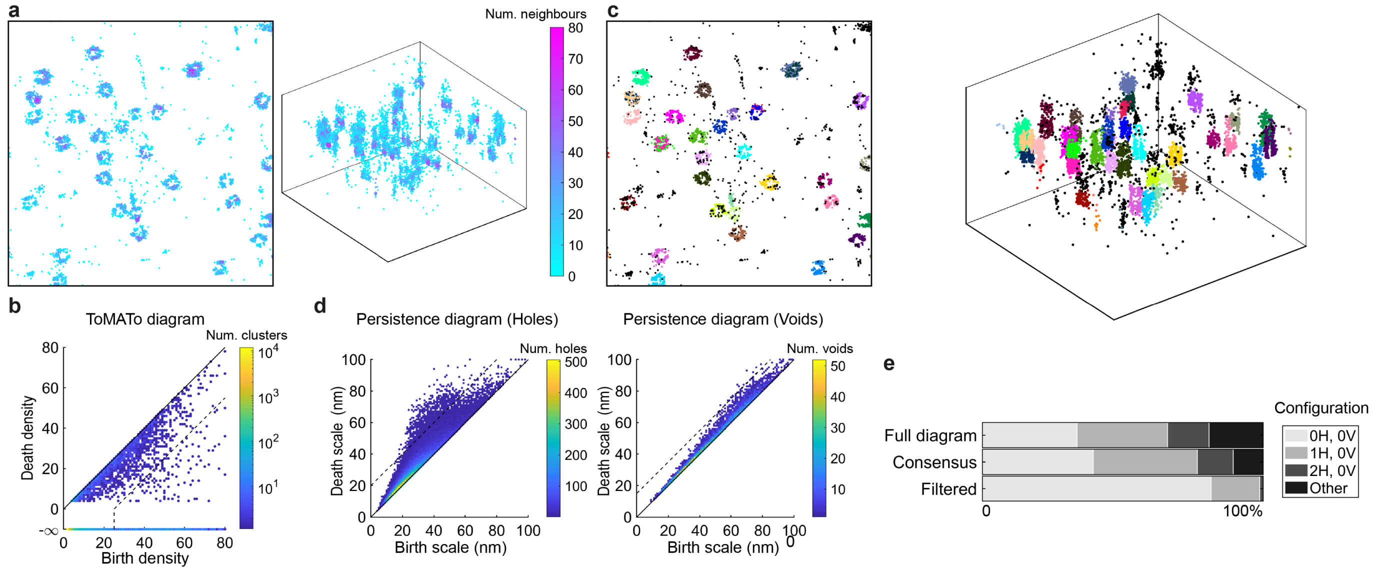

Fig. S16. Clustering and topological analysis of nuclear pore component Nup107 in 3D. (**a**) Density estimate was calculated using a search radius of 50 nm. Representative cropped field of view shown as a projection and 3D scatter-plot. (**b**) ToMATo diagram where a persistence threshold of 25 nm was applied (dotted line). (**c**) Result of ToMATo clustering to segment nuclear pore complexes in 3D. Clusters were filtered by number of detections $(20 - 400)$ and volume $(4/3\pi \times 40^3 - 4/3\pi \times 150^3nm^3)$. (**d**) Persistence diagrams for 2D and 3D topological features. Thresholds of 20 nm and 15 nm were chosen for holes and voids respectively. (**e**) Percentage of clusters with specified topological configuration. This was calculated using either the full diagram, the sub-sampled consensus, or the sub-sampled consensus with agreement > 90%.
